# Spinal Mechanisms May Provide a Combination of Intermittent and Continuous Control of Human Posture: Predictions from a Biologically Based Neuromusculoskeletal Model

**DOI:** 10.1371/journal.pcbi.1003944

**Published:** 2014-11-13

**Authors:** Leonardo Abdala Elias, Renato Naville Watanabe, André Fabio Kohn

**Affiliations:** Biomedical Engineering Laboratory, Escola Politécnica, University of Sao Paulo, Sao Paulo, Brazil; University of Southern California, United States of America

## Abstract

Several models have been employed to study human postural control during upright quiet stance. Most have adopted an inverted pendulum approximation to the standing human and theoretical models to account for the neural feedback necessary to keep balance. The present study adds to the previous efforts in focusing more closely on modelling the physiological mechanisms of important elements associated with the control of human posture. This paper studies neuromuscular mechanisms behind upright stance control by means of a biologically based large-scale neuromusculoskeletal (NMS) model. It encompasses: i) conductance-based spinal neuron models (motor neurons and interneurons); ii) muscle proprioceptor models (spindle and Golgi tendon organ) providing sensory afferent feedback; iii) Hill-type muscle models of the leg plantar and dorsiflexors; and iv) an inverted pendulum model for the body biomechanics during upright stance. The motor neuron pools are driven by stochastic spike trains. Simulation results showed that the neuromechanical outputs generated by the NMS model resemble experimental data from subjects standing on a stable surface. Interesting findings were that: i) an intermittent pattern of muscle activation emerged from this posture control model for two of the leg muscles (Medial and Lateral Gastrocnemius); and ii) the Soleus muscle was mostly activated in a continuous manner. These results suggest that the spinal cord anatomy and neurophysiology (e.g., motor unit types, synaptic connectivities, ordered recruitment), along with the modulation of afferent activity, may account for the mixture of intermittent and continuous control that has been a subject of debate in recent studies on postural control. Another finding was the occurrence of the so-called “paradoxical” behaviour of muscle fibre lengths as a function of postural sway. The simulations confirmed previous conjectures that reciprocal inhibition is possibly contributing to this effect, but on the other hand showed that this effect may arise without any anticipatory neural control mechanism.

## Author Summary

The control of upright stance is a challenging task since the objective is to maintain the equilibrium of an intrinsically unstable biomechanical system. Somatosensory information is used by the central nervous system to modulate muscle contraction, which prevents the body from falling. While the visual and vestibular systems also provide important additional sensory information, a human being with only somatosensory inputs is able to maintain an upright stance. In this study, we used a biologically-based large-scale neuromusculoskeletal model driven only by somatosensory feedback to investigate human postural control from a neurophysiological point of view. No neural structures above the spinal cord were included in the model. The results showed that the model based on a spinal control of posture can reproduce several neuromechanical outcomes previously reported in the literature, including an intermittent muscle activation. Since this intermittent muscular recruitment is an emergent property of this spinal-like controller, we argue that the so-called intermittent control of upright stance might be produced by an interplay between spinal cord properties and modulated sensory inflow.

## Introduction

The maintenance of upright quiet stance is a challenging task for the central nervous system (CNS), and the objective is to achieve the control of an intrinsically unstable biomechanical system under the effect of gravity. Posture control is a position control problem in which the CNS, leg muscles, and different sensory systems (e.g., the muscle proprioceptors) interact to maintain the horizontal projection of the centre of mass (COM) within a region bounded by the feet (for a review of the basics of posture control, see [1]). Sensory systems, such as the vestibular, visual, and somatosensory, play a significant role in the aforementioned motor task, so that disorders in any of these systems may lead to postural instability [2], [3].

A conceptual question under debate in the literature concerns the manner the CNS controls upright stance in adults (i.e., human beings who already learned to walk). Some researchers argue in favour of a negative-feedback continuous control, with the leg muscles reflexively activated in response to drifts or perturbations away from an equilibrium position [4]–[6]. Conversely, others suggest that an anticipatory (feedforward) mechanism is necessary to explain some findings, such as the low feedback gains observed in some experiments [7]–[9]. More recently, the view that human upright stance is controlled by an intermittent mechanism has grown [10]–[15]. The latter is based in part on results from an experiment involving the manual balancing of a virtual inverted pendulum controlled by a subject through a computer joystick [13]. Additionally, other recent studies showed that motor units (MUs) of the Medial Gastrocnemius (MG) muscle exhibited an intermittent recruitment during upright quiet stance with a pattern closely linked to COM and centre of pressure (COP) fluctuations [14], [16]. It is noteworthy that the previously referred papers used a wide-sense/physiological conceptualisation of intermittent postural control, which is understood as one exhibiting discrete (bursty) actions of the neuronal controller, producing ballistic-like (phasic) muscle activations. In addition to this more qualitative/physiological view of intermittency, there is a strict, quantitative, definition of intermittent control that has also been used to analyse motor control problems, such as stick balancing and postural control [17]. In the present study we will adopt the former (physiological) view of intermittency.

From a theoretical standpoint, mathematical models have been developed to describe and to investigate human postural control. These models explicitly incorporate the above-mentioned control strategies hypothesised to be adopted by the CNS during upright standing control, but do not identify which part of the CNS the control resides, e.g. cortex, brainstem, cerebellum, spinal cord. For instance, [4], [5], [18]–[20] represented the postural control system as a negative-feedback continuous control system, so that proprioceptive information of body position and velocity were fed back to the CNS. Alternatively, the models proposed by [21]–[23] adopted a continuous predictive control system, while others [11], [12], [24], [25] represented the control of posture as an intermittent control system. Despite these fundamental differences, all these models were based on a control engineering framework, whereby the whole system was simplified and the CNS was represented by a PD/PID (proportional-derivative/proportional-integral-derivative) controller, an optimal continuous controller or an intermittent controller that activated the muscles in discrete bursts. Despite their advantages of a relative simplicity and the power of explanation, it is not easy to translate their results in terms of the underlying physiological mechanisms involved in the control of human posture.

The present study aims at providing a complementary approach to those mentioned above in that the fundamental focus is on biology (anatomy and physiology). A large amount of physiological knowledge, from the behaviour of neuronal ionic channels to the dynamics of muscle contraction, was funnelled into a large-scale mathematical model of the nervous, muscular, and biomechanical systems involved in posture control. In this vein, a biologically based large-scale neuromusculoskeletal (NMS) model was developed and used to investigate the problem of postural control from a more neurophysiological standpoint. The model encompasses: i) a spinal neuronal network, which includes conductance-based models of both 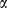 motor neurons (MNs) and interneurons (INs); ii) Hill-type muscle models to represent the viscoelastic properties of the Soleus (SO), MG, Lateral Gastrocnemius (LG), and Tibialis Anterior (TA) muscles; iii) models of both muscle spindle and Golgi tendon organ (GTO); iv) afferent fibres providing Ia, II, and Ib feedback; and v) an inverted pendulum model, which is a first approximation of upright quiet stance [26]. Here the proprioceptive feedback is provided by muscular proprioceptors (i.e., spindles and GTOs), since they seem to be largely responsible for the position sense of the limb [27], [28]. The first hypothesis of the present study is that stance control might be properly achieved by a spinal-like controller (SLC, approximated here by the developed NMS model) based on a proprioceptive feedback. An associated second hypothesis is that the activation of leg muscles by this SLC is a continuous process yielding the maintenance of human body equilibrium.

Another relevant issue related to postural control that the present study analyses is related to recent experimental findings of a “paradoxical” behaviour of the calf's muscle fibre lengths during postural sway [29], [30]. The behaviour was called “paradoxical” because the muscle fibre lengths were negatively correlated with COM/COP displacements [31]. These studies proposed that reciprocal inhibition from antagonistic (TA) Ia afferents might be responsible for this unexpected motor behaviour. With the availability of the detailed NMS model employed in the present study, the correlation between muscle fibre length and COM/COP displacements could be tackled without much difficulty. Therefore, a third hypothesis of the present study is that a model structure without the reciprocal inhibition pathway (from TA Ia afferents to Triceps Surae MNs) would exhibit positive correlation coefficients between muscle fibre length and COM/COP displacements. A complement of this hypothesis is that the reciprocal inhibition neural circuit may increase the probability of occurrence of the “paradoxical” relation mentioned above. This combined third hypothesis is, therefore, evaluated here using the complex NMS physiological model in order to verify a hypothesis put forward by previous authors on the basis of heuristics and experimental results from humans [29]–[31].

To the best of our knowledge, this is the first study to address the control of an intrinsically unstable neuro-biomechanical system associated with the maintenance of human quiet standing by means of a complex large-scale system mostly based on known physiology. However, others have also used biologically based reductionist models of the NMS system to investigate how the CNS controls other motor tasks [32], [33]. A minor part of this material was already published as conference abstracts [34], [35].

## Results

### Model Validation in Terms of Postural Control

**Figure 1:**
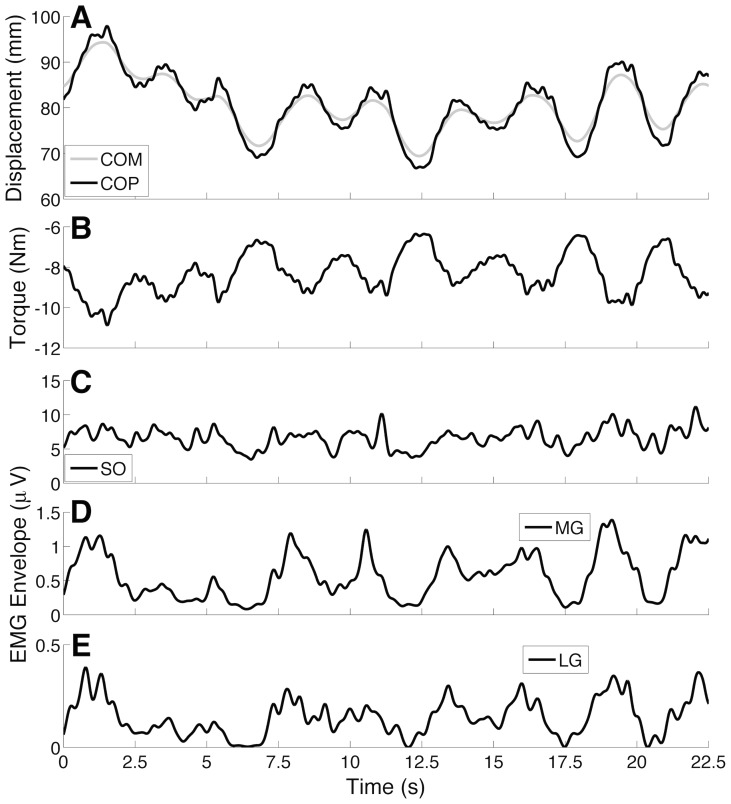
Neuromechanical outputs of the postural control model (*Model 2*) for a typical simulation. (A) Anteroposterior centre of mass (COM; gray curve) and centre of pressure (COP; black curve) displacements. (B) Muscular torque produced during postural sway. The negative value represents a plantar-flexion torque produced by the leg muscles (activation of the Triceps Surae muscle group) (C-E) Electromyogram (EMG) envelopes from Soleus (SO), Medial Gastrocnemius (MG), and Lateral Gastrocnemius (LG) muscles.

Typical biomechanical and neuronal outputs of the NMS model are presented in Figure 1. The model's responses resemble qualitatively those frequently reported in postural control studies (e.g., [9], [29], [36]). Irrespective of model structure (i.e., *Model 1* or *Model 2* - see Methods for details), the inverted pendulum leaned about 5 deg forward (equilibrium point), so that COM and COP displacements oscillated around a basal value of 80 mm (see Figure 1A). The basal plantar-flexion torque (negative torque) was 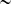
10% of the maximum isometric torque produced by the model. One can notice that COM and COP (Figure 1A) oscillated in anti-phase with respect to the muscle torque (Figure 1B), i.e. when the body leaned forward from its equilibrium position the plantar-flexion torque increased (more negative). Conversely, muscle activations (EMG envelopes in Figure 1C-E) were modulated approximately in phase with postural sway. In the simulations, TA muscle was silent during postural sway (not shown).

**Figure 2:**
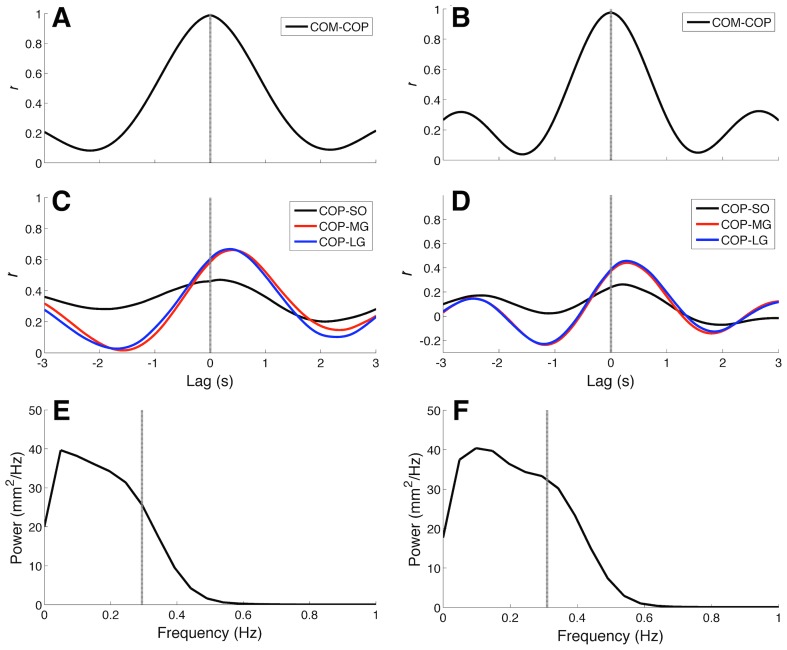
Cross-correlation functions and centre of pressure (COP) power spectra for typical simulations carried out on *Model 1* (graphs A, C, and E) and *Model 2* (graphs B, D, and F). (A-B) Cross-correlation functions between centre of mass (COM) and COP. Note that for both models, cross-correlation peaks occurred at zero lag (dashed lines). (C-D) Cross-correlation functions between COP and muscle electromyograms (EMGs). Black, red, and blue curves represent cross-correlation functions for Soleus (SO), Medial Gastrocnemius (MG), and Lateral Gastrocnemius (LG), respectively. Irrespective of the model structure, there was a lag of about 300 ms between COP and EMG envelopes from the three muscles. (E-F) COP power spectra. Dashed line represents the 50% power frequency (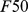). It is noteworthy that for *Model 2* there was a broader bandwidth in comparison to *Model 1*.

A quantitative analysis was performed to validate the model with respect to the available data from the literature. Typical time-domain metrics were calculated from the COP time series and compared to data from normal subjects and vestibular loss patients standing on a force plate without visual information (see Table 1). Root mean square (RMS) and mean velocity (MV) of simulated COP were higher than the values observed experimentally in normal subjects, but compatible with data from vestibular loss patients. Another quantitative validation was based on a cross-correlation analysis performed between the COM and COP time series (Figure 2A-B), as well as between COP and EMG envelopes (Figure 2C-D). COM and COP were highly correlated (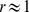) at lag zero. COP and EMG envelopes were positively correlated with the correlation peak occurring at a positive lag. Correlation coefficients (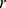) and cross-correlation peak lag values were compatible with experimental data from healthy subjects (see Table 1). In general, correlation coefficients were higher for Gastrocnemii in comparison to the SO, and muscles' activations (EMGs) were advanced by approximately 200–300 ms in relation to the postural sway (COP). The 50% power frequency (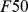) estimated from the COP power spectrum (see Figure 2E-F) resulted quite similar to the value from healthy subjects (see Table 1). COP power spectra of both model structures were limited to 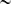
1 Hz.

**Table 1:** Centre of Pressure (COP) metrics and cross-correlation analysis between COP and electromyogram (EMG) obtained from the two model structures in comparison with experimental data reported in the literature.

Variable (Unit)	Model 1 (*n* = 3)	Model 2 (*n* = 3)	Experimental Data (*n*) [Ref.]
COP RMS (mm)	9.24 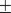 0.92	9.75 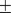 0.99	3.82 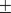 1.54 (20) [42]
			14.90 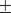 11.50 (10) [3]
COP MV (mm/s)	9.14 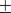 0.93	10.83 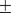 0.86	6.72 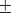 2.18 (20) [42]
			60.20 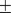 59.90 (10) [3]
COP 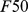 (Hz)	0.29 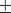 0.01	0.31 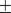 0.03	0.30 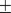 0.09 (20) [42]
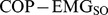 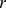 (−)	0.39 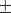 0.11	0.15 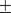 0.13	0.57 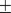 0.20 (16) [43]
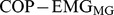 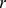 (−)	0.63 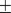 0.11	0.40 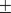 0.09	0.71 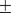 0.12 (16) [43]
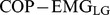 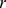 (−)	0.62 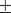 0.16	0.40 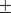 0.08	0.59 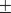 0.20 (16) [43]
			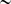 0.45 (7) [9]
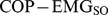 Peak Lag (ms)	213.50 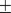 25.50	247.80 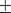 73.60	200 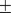 20 (16) [43]
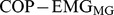 Peak Lag (ms)	351.50 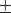 50.40	322.30 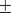 25.80	190 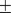 40 (16) [43]
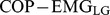 Peak Lag (ms)	311.70 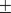 86.20	268.80 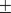 49.40	210 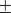 30 (16) [43]
			240–270 (7) [9]

*Model 1* is the postural control model without reciprocal inhibition, whereas *Model 2* is the complete postural control model (see Methods for details concerning model structures). Data are expressed as Mean 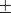 Standard Deviation. 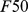 is defined as the frequency at which 50% of the total power of the COP is confined (see Data Analysis). Values of 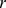 were measured as the peak values of the cross-correlation functions between COP and the EMGs of the Triceps Surae (TS) muscles (i.e., SO, MG, and LG). Data from [9], [42], [43] were obtained from healthy young subjects standing on a stable surface without visual feedback, while [3] provided data from vestibular loss patients standing on a stable surface without visual feedback.

**Figure 3:**
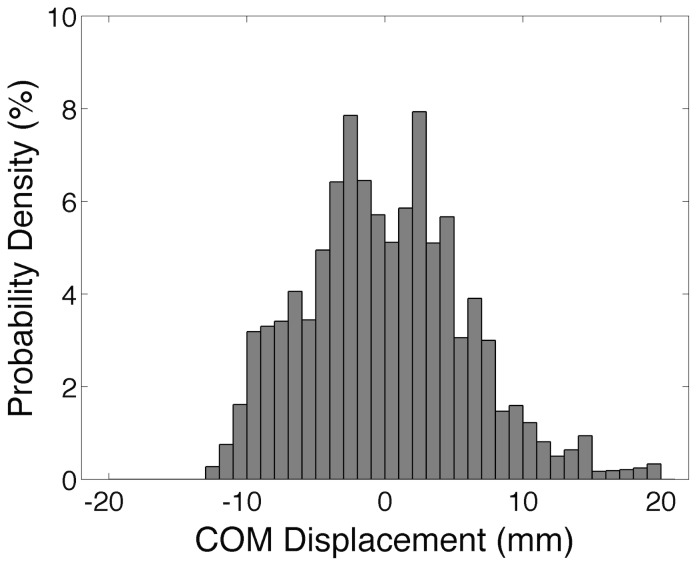
Pooled histogram of centre of mass (COM) displacements from the simulations performed on *Model 2*. The mean values of COM displacement (mean equilibrium positions of the inverted pendulum) were subtracted from each simulated COM time series so that the data from different simulation runs could be pooled and hence plotted in the same graph. Note that the histogram exhibits a clear bimodal shape (see text for details).

A final quantitative validation was based on the pooled histogram of COM displacements (1-mm bins) as shown in Figure 3 (data are from the simulations of *Model 2*). The histogram shape was bimodal, with two peaks around the equilibrium position of the inverted pendulum (value 0 in the abscissa). The Jarque-Bera goodness-of-fit test was applied to verify if this histogram could be fitted by a typical Gaussian probability density function [11]. The null-hypothesis (the histogram comes from an unimodal Gaussian function) was rejected (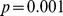). The same result was obtained for *Model 1*.

### Intermittent Recruitment of the Motor Units

**Figure 4:**
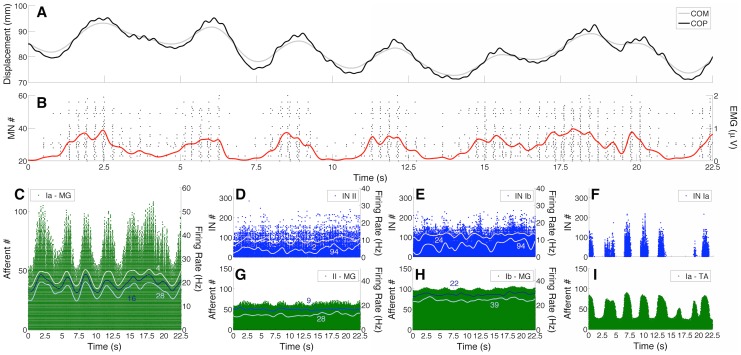
Intermittent recruitment of Medial Gastrocnemius (MG) motor units (MUs) and modulation of proprioceptive feedback (typical simulation performed on *Model 2*). (A) Centre of mass (COM; gray curve) and centre of pressure (COP; black curve) displacements. (B) Raster plots (black dots) of 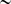
40 MG MUs intermittently recruited during quiet standing. Red curve represents the global MG electromyogram (EMG) envelope. Note the ballistic-like (phasic) activation of this muscle during postural sway. (C) Raster plots for the population of Ia afferents from the MG muscle. Note the clear modulation in the recruitment of primary afferents. In addition, continuous curves show the instantaneous firing rate (estimated by a Gaussian kernel convolved with the spike trains) for three Ia afferents (4, 16, 28). (D-F) Raster plots for group II excitatory interneurons (INs), Ib inhibitory INs, and Ia inhibitory INs. Continuous curves in panels D and E represent the instantaneous firing rate for two type-specified INs (2 and 94 for group II INs; 24 and 94 for Ib INs). (G-I) Raster plots for type II afferents from MG muscle spindle, Ib afferents from MG muscle spindle, and Ia afferents from TA muscle spindle. Continuous curves in panels G and H represent the instantaneous firing rate of two type-specified afferent fibres (9 and 28 for type-II afferents; 22 and 39 for Ib afferents).

**Figure 5:**
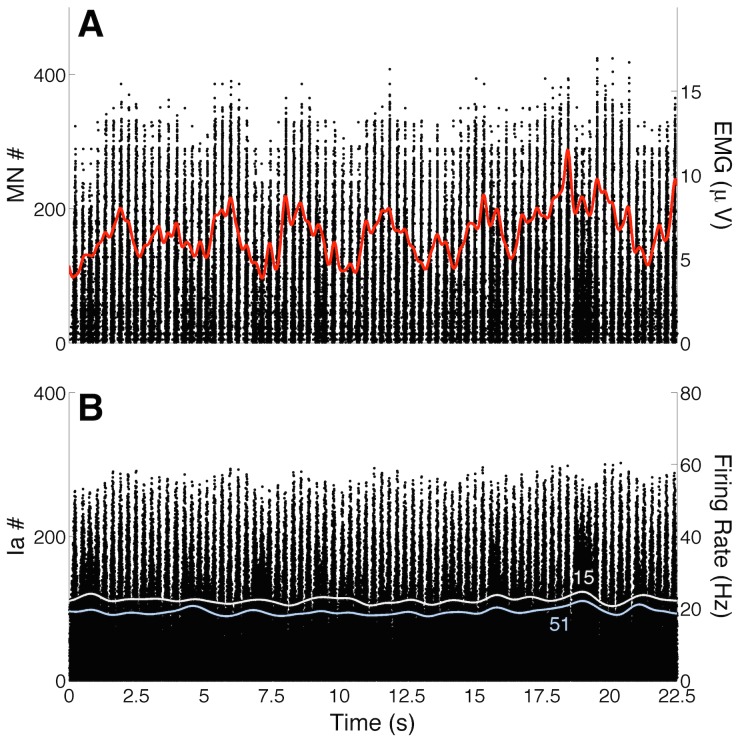
Typical behaviour of Soleus (SO) motor units (MUs) and Ia afferents. (A) Raster plots (black dots) and SO electromyogram (EMG) envelope (red curve). Note that the SO muscle has an approximately steady activation during postural sway (not too much recruitment and de-recruitment). (B) Raster plots from SO muscle spindle Ia afferents and instantaneous firing rate for two selected afferents (continuous curves for afferents 15 and 51). Note the small modulation in Ia afferent recruitment and firing rate.

Figures 4 and 5 show how the spike trains from spinal MNs, INs, and afferent fibres were modulated during postural sway. An interesting qualitative finding was that MUs from the MG muscle were intermittently recruited/de-recruited as the inverted pendulum swayed forward/backward (Figure 4B). This intermittent pattern of MU recruitment was similar for the LG muscle (not shown), but less evident for the SO muscle (see Figure 5A). The degree of intermittency for the MG and SO MUs was quantified by the activation ratio (see [16] and Methods for details). The median (range) activation ratios calculated for 90 randomly selected MG MUs (30 MUs were chosen per simulation) from *Model 1* and *Model 2* were 0.69 (0.44–0.80) and 0.65 (0.47–0.81), respectively. For 90 randomly selected SO MUs the activation ratios were 0.97 (0.75–1) and 0.96 (0.79–1) for *Model 1* and *Model 2*, respectively. Because of these results, the MG and LG muscles were considered to have ballistic-like activations (see EMG envelopes in Figures 1D-E and 4B), while the SO muscle was mostly tonically (continuously) active during the maintenance of an upright posture (see Figures 1C and 5A).

In order to quantify the intermittent recruitment of MG MUs, the interval between successive recruitments was computed for a subset of 30 randomly chosen MUs (10 MUs were chosen per simulation). In accordance with the method used by [14], intermittent recruitment was considered if a given MU was discharging at a rate lower than 4 Hz (i.e., interspike intervals higher than 250 ms). For *Model 1*, 899 intervals of 30 MG MUs were evaluated and the mean (modal) interval between successive recruitments was equal to 511 (274) ms [i.e. a mean (modal) rate equal to 1.96 (3.65) Hz]. Similarly, for *Model 2*, 846 intervals of 30 MG MUs had a mean (modal) value of 505 (277) ms [1.98 (3.61) Hz]. Therefore, both model structures produced a similar intermittent recruitment pattern on MG MUs. A low number of LG MUs was recruited (less than 30) and the SO MUs were mostly tonically active during the simulation of postural control (see the activation ratios in previous paragraph), hence the intermittency of the MUs from these muscles were not quantitatively evaluated here.

Panels C-I in Figure 4 and panel B in Figure 5 show typical results of how proprioceptive feedback (encompassing afferent fibres and spinal INs) was modulated during sway (*Model 2* was used for this simulation). The activity of the Ia afferents from the MG muscle (Figure 4C) was highly modulated, following approximately the COM/COP displacement (note the firing rate modulation for three different Ia afferents, as indicated by the thin lines). Since there was little variation in the MG muscle torque (RMS value 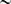
2.50% of the maximum MG muscle torque) and the mean MG muscle fibre length was maintained at an approximately steady value (i.e., there was little change in the static component of the muscle fibre length - see below), the activities of Ib and type II afferents were minimally modulated (see panels G and H in Figure 4). The proprioceptive pathways responsible for the reciprocal inhibition were also highly modulated during postural sway (see panels F and I in Figure 4). Inhibitory Ia INs discharged phasically when the inverted pendulum swayed backward and this contributed to a decrease in the ankle joint torque generated by the plantar flexor muscles. Conversely, Ia afferents from the SO muscle spindles were poorly modulated in the posture control task (see Figure 5B).

**Figure 6:**
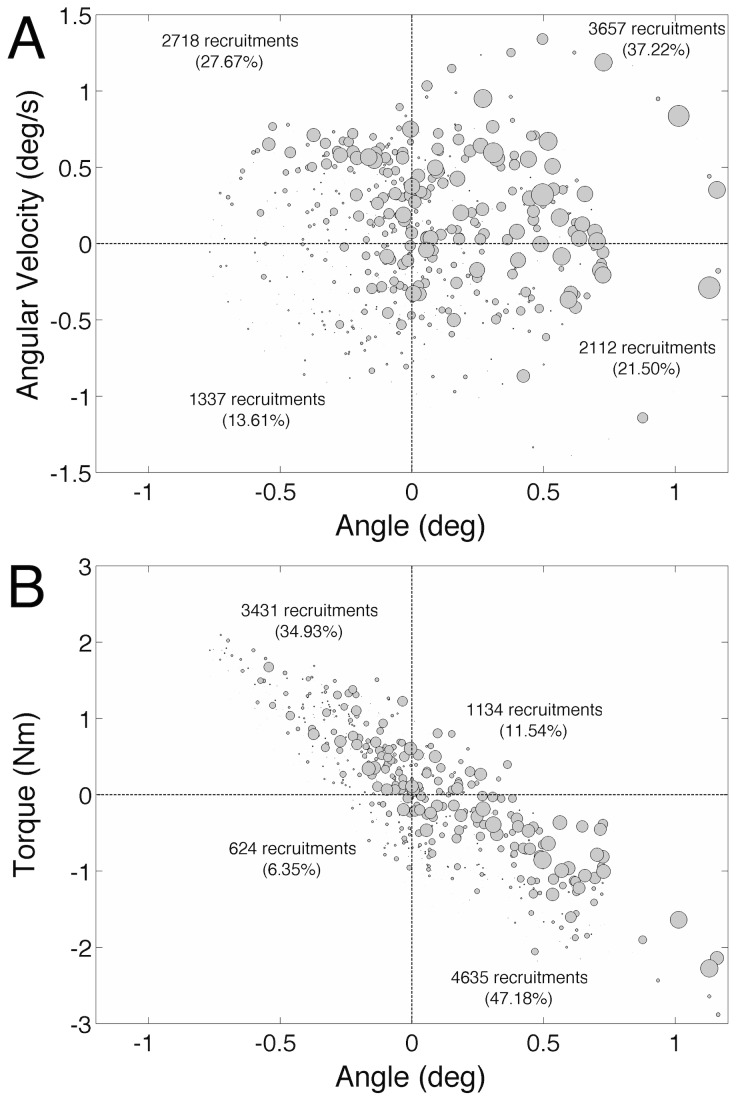
Recruitment phase plots. (A) Phase plot showing the recruitment of Medial Gastrocnemius (MG) motor units (MUs) in angle-velocity bi-dimensional graph. The point (0,0) is a reference due to the subtraction of the mean value of each variable obtained in the simulations. Circle diameters are proportional to the number of recruited MUs (see Methods). It is noteworthy that MUs seem to be preferentially recruited in the first quadrant, i.e., when the pendulum is leaning forward as it comes from a backward position. (B) Phase plot showing the recruitment of MG MUs in angle-torque bi-dimensional graph. Again, the point (0,0) is a reference that was used because each simulation produced slightly different mean torques and angles. Note that MG MUs are mostly activated in the fourth quadrant, i.e., when the pendulum is leaning forward and the plantar-flexion torque produced by Triceps Surae (TS) is higher than the mean.

The intermittent recruitment of MG MUs was evaluated on the basis of two phase plots that relate angular velocity and muscle torque with ankle angle data obtained from the postural control model (Figure 6). Figure 6A shows that most of the MG MUs (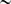
60%) were recruited when the inverted pendulum was leaning forward from its equilibrium position irrespective of its velocity (first and fourth quadrants of the angle-velocity phase plots). Nonetheless, a large number of MUs (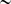
28%) were recruited when the inverted pendulum was at a backward position but with a positive velocity (second quadrant), i.e., the pendulum was starting to return to a forward position. Similarly, most of the MG MUs (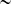
50%) were recruited when the pendulum was leaning forward and producing a higher plantar flexion torque (fourth quadrant in the Figure 6B), i.e., the pendulum was at a forward position and decelerating. Almost 35% of the MUs were recruited when the pendulum was at a backward position and with a lower (more positive than the mean value) plantar flexion torque. In general, the discharges in the first and third quadrants were mainly involved in the generation of a basal torque, while the discharges in the second and fourth quadrants represented the phasic corrective torque control produced by the MG muscle.

### Effect of Reciprocal Inhibition

Two model structures were adopted to investigate the effect of the reciprocal inhibition pathway on postural control (see Methods for details). According to the data presented in the previous section, there were no dramatic differences in the time- and frequency-domain metrics, as well as in the MU recruitments. The COP power spectrum calculated from the model without reciprocal inhibition (*Model 1*) was slightly narrower than that from *Model 2*. In addition, correlation coefficients between COP and EMG envelopes were lower when the reciprocal inhibition pathway was included in the model. Nonetheless, both model structures produced fluctuating outputs that resembled experimental data, suggesting that reciprocal inhibition from TA Ia afferents is not a strict requisite for the control of upright standing.

**Figure 7:**
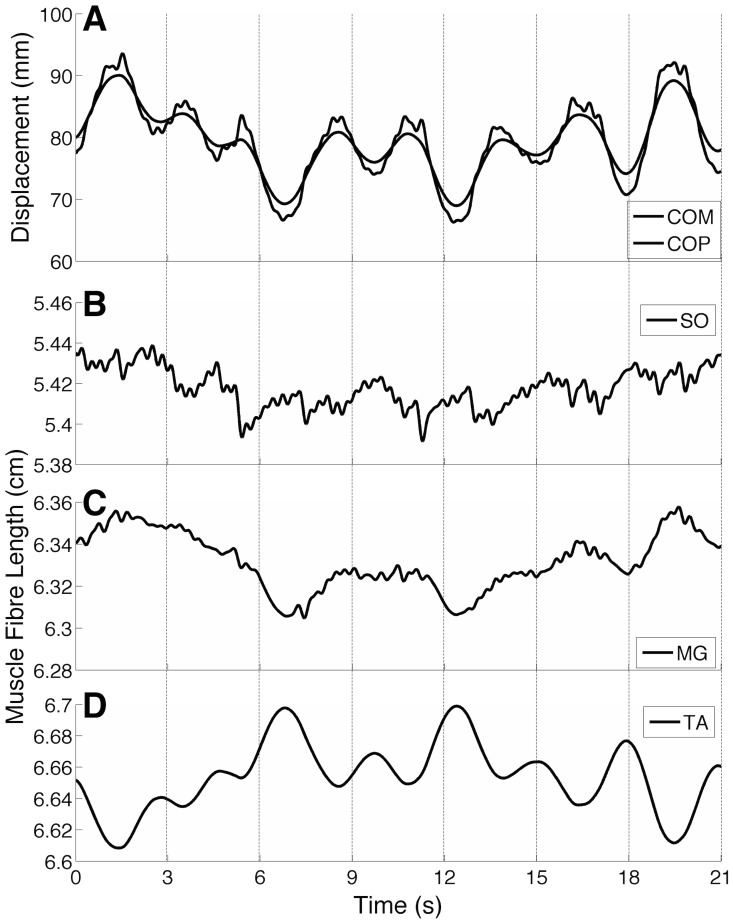
Modulation of muscle fibre lengths during postural sway (typical simulation performed on *Model 2*). (A) Centre of mass (COM; gray curve) and centre of pressure (COP; black curve) displacements. (B-D) Muscle fibre lengths from Soleus (SO), Medial Gastrocnemius (MG), and Tibialis Anterior (TA) muscles. Lateral Gastrocnemius (LG) was not shown here because its behaviour is quite similar to that of the MG muscle. Dashed lines represent the 3-s duration windows used to perform the correlation analysis between COM and muscle fibre lengths (see Methods for details and Figure 8).

**Figure 8:**
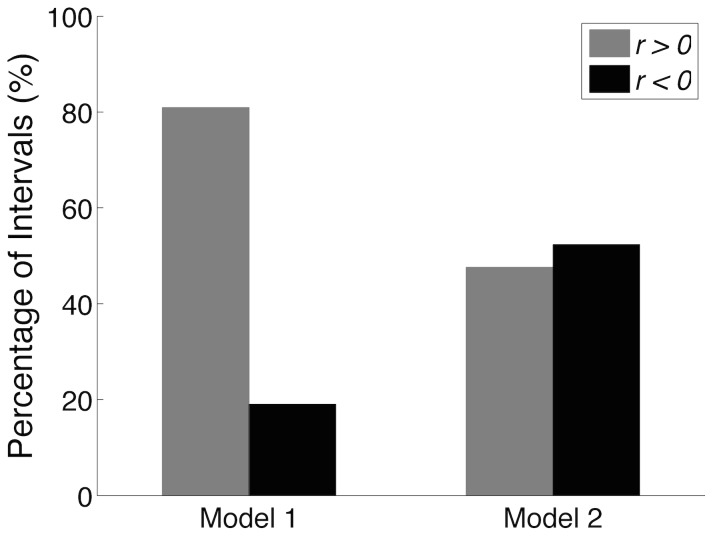
Correlation analysis between centre of mass (COM) displacement and Soleus (SO) muscle fibre length. Each bar represents the percentage of intervals (3-s duration windows) with positive (gray) and negative (black) correlation coefficients for each model structure. For *Model 1*, 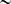
80% of intervals (17/21) had positive correlation coefficients, while for *Model 2* this proportion was lower than 50% (10/21). Statistically significant difference was observed between the results from the two model structures (*Model 1* vs. *Model 2*, 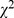-test, 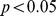). These results suggest that reciprocal inhibition may contribute significantly to the “paradoxical” behaviour (negative correlation between COM and muscle fibre length) reported in the literature [29]–[31].

In the present study, we tested the hypothesis that reciprocal inhibition may be responsible for the negative correlation between muscle fibre length and COM/COP displacement [29]–[31]. This was tested by performing a correlation analysis between COM displacements and muscle fibre lengths for the SO, MG, and TA. Typical signals representing these variables are shown in Figure 7. For this typical simulation (*Model 2*) one can notice that MG muscle fibre length was positively correlated with COM, while TA muscle fibre length and COM displacement were negatively correlated. The latter results are a typical “orthodox” behaviour that has been shown for some healthy subjects during upright quiet standing [29]. For the SO muscle, a more quantitative analysis was performed (see Figure 8). Correlation analysis between SO muscle fibre length and COM displacement was performed on 3-s windows (see dashed vertical lines in Figure 7) according to the method adopted in [29]. Correlation coefficients calculated from *Model 1* and *Model 2* were pooled into two groups: positively correlated (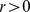) and negatively correlated (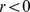). For *Model 1* most of the intervals (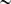
80%) showed a positive correlation between SO muscle fibre length and COM displacement (i.e., “orthodox” behaviour), whereas for *Model 2* the number of negatively correlated intervals increased to 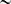
50%. 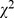-test revealed a statistically significant difference (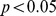) between the responses of the two model structures. This suggests that, at least for the SO muscle, reciprocal inhibition might be involved in the genesis of the “paradoxical” behaviour of muscle fibre length. Nonetheless, a small percentage of negatively correlated intervals (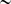
20%) might also be generated in the absence of any reciprocal inhibition from antagonistic Ia afferents.

## Discussion

A large-scale NMS model was applied in the present study to the problem of controlling upright standing in humans. A different feature of this approach in comparison with most of the previous studies in the literature (e.g., [4]–[6], [10]–[12], [18], [19], [22]–[24]) is that the structure and behaviours of each element were based on known physiology, anatomy, and biomechanics encompassing important parts of the postural control system. Therefore, the control strategy employed by the modelled CNS emerged from the interaction between several neuromechanical elements involved. While the overall mechanisms that control the inverted pendulum sway are beyond an analytical understanding, the increased biological realism provides important clues regarding some putative neurophysiological mechanisms underlying the posture control task. In the following sections, the results presented earlier shall be discussed with respect to relevant experimental findings reported in the literature.

### Postural Control in Quiet Standing

The results presented here showed that a SLC was effective in maintaining the equilibrium of an intrinsically unstable biomechanical system (see Figure 1 for an illustrative example). This leads to the first contribution of this study, which is to support the hypothesis that human upright quiet standing may be properly controlled by spinal mechanisms, for example, without any cortical involvement. This view is consistent with several studies, which suggest that cortical control is decreased or may be absent when a motor task is well trained (e.g., [30]–[40]). Normal quiet postural control, under no special restrictions (as standing on a narrow beam or during dual tasks), is certainly a candidate for a well-trained task that would not require cortical control. Since the scope of the present study is limited to the investigation of neurophysiological mechanisms underlying the control of quiet standing, the assumed lack of supraspinal neural structures should not limit the ensuing interpretations. A separate section below (see Model Limitations and Future Research) presents and discusses the limitations both of the modelling as well as the conclusions derived from the simulations.

Time- and frequency-domain metrics obtained from the NMS model were compatible with experimental data (see Table 1). The equilibrium values of the inverted pendulum (mean angle, torque, and COM/COP displacements) varied slightly both within and between simulations due to programmed (randomised) changes in system configuration. Between-simulation changes occurred due to changes in neuronal connectivities and intrinsic properties (e.g., action potential thresholds) that were randomly attributed at the beginning of each simulation [41]. On the other hand, within-simulation variations were mainly related to the number of recruited MUs, which varied stochastically due to neuronal noise. Therefore, the mean equilibrium position of the body depends on the overall instantaneous configuration of the postural control system. The analysis of the COP time series showed that the model was less stable (i.e., presented a larger postural oscillation) than healthy young subjects [42]. Notwithstanding, simulated data were compatible with those from vestibular loss subjects standing on a stable surface without visual information [3]. As a consequence, the simulation results reinforce that the increased postural oscillation observed in patients may be due to the lack of other sensory inputs providing information to the CNS, such as vestibular and visual sources. Or, in other words, the proprioceptive feedback gain in such patients is not sufficient to replace the other missing sensory feedback modalities. Interestingly, the variability observed in the simulated postural sway was exclusively generated by the variability in sensory afferents and descending commands, which results in random fluctuations of motoneuronal discharges. Therefore, it is predicted that most of the biomechanical variability (sway) observed during upright standing has a neuronal origin and is less influenced by internal disturbing forces (e.g., heartbeats and respiration) as proposed elsewhere [4], [6], [8], [11], [25].

The cross-correlation analysis (Figure 2) showed that EMGs from the Triceps Surae (TS) muscles were positively correlated with postural sway (as measured by the COP). Simulation results are in accordance with experimental data that showed higher correlation coefficients between EMGs from Gastrocnemii and COP [9], [43]. Additionally, the time lags between COP and EMGs were within a range of 200–300 ms, which is also compatible with experimental data [9], [43]. This is in some sense a remarkable result that emerged from the NMS model since the sum of the afferent and efferent action potential propagation delays is much smaller than this time lag between COP and EMG. Albeit qualitative, or semi-quantitative, this is a relatively strong sign that the model was able to capture at least a part of the overall system dynamics. In [9] it is argued that the existence of this lag between the mechanical and neuronal responses would be due to an anticipatory action of the neuronal controller, i.e., the postural control is mediated by a feedforward mechanism. However, theoretical and computer simulation studies [19] showed that even in the absence of any feedforward mechanism, lags between neuromechanical signals may be obtained depending on the parameters of the continuous feedback system and stochastic features of the input signals. The results presented here corroborate the latter view, since no feedforward mechanism was incorporated into the NMS model.

Another experimental finding from human postural control studies that was reproduced by the model was the bimodal distribution of COM displacements (see Figure 3). In [11] most of the data from young subjects exhibited double-peaked histograms of COM displacements with a local minimum in between (see their Figure 5). The authors of the above-mentioned study [11] showed that the bimodal distribution of COM displacements was only obtained when they represented the postural control system by a mixture of both continuous and intermittent (with a phasic controller operating at a 3–4 Hz burst rate) control mechanisms. A continuous postural control model produced unimodal Gaussian-like histograms. Therefore, they argued that the postural control in humans is not mediated exclusively by a continuous control mechanism. Our results corroborate this proposal. However, in our approach the control structure (e.g., continuous, intermittent, or a mixture of both) was not imposed *a priori*. The mixture of continuous (SO motor nucleus and muscle fibres) and intermittent (mostly Gastrocnemii motor nuclei and muscle fibres) control behaviours was a result of the interactions of the several interconnected neuromusculoskeletal elements of the model and their respective dynamics. Further discussion on the issue of continuous and intermittent control mechanisms operating during postural control shall be presented in a separate section below.

It is worth mentioning that a key parameter for stabilizing the inverted pendulum model was the constant level of the fusimotor activity that adjusted the sensitivity of muscle spindles for each simulation. Without a proper value for both static and dynamic fusimotor activities the pendulum fell at the beginning of the simulation. As previously mentioned, some parameters were randomly distributed in each simulation run [41], hence producing a different set of initial conditions in different runs. This explains why the mean values of fusimotor activities vary (slightly) across the different simulation trials (see Methods). In the context of human postural control, the dependence of an effective control upon the fusimotor activity suggests that the CNS must properly set the muscle spindle sensitivity for the performance of the task [27], [44], [45].

Another point that should be stressed is that the NMS model was not stabilised without the neuronal activity, i.e., the pendulum fell when the SLC was turned off. This is compatible with the current view that the viscoelastic properties of the muscles around the ankle joint are not sufficient to control upright standing [8], [46]–[48]. In the model, the intrinsic passive ankle joint stiffness was about 70% of the critical toppling torque (see Methods for details), which is in accordance with the experimental estimates at ankle joint rotation angles similar to those obtained in our model (about 0.60 deg on average) [46], [48]. The adoption of a constant passive stiffness is a typical simplification (see for instance [5], [11]) that was also adopted in the present study. In an analytical study [49], the conclusion was that the feedback provided by the muscle spindles and GTOs is not sufficient to stabilize an inverted pendulum representing the human body. However, the authors did not considered any passive mechanism at the joint level and, hence, the total torque was generated by the active neural controller. Here, as the passive properties were included, the demand of the CNS was about 30% of the necessary stabilizing torque, which is compatible with the experimental estimates [11], [46].

### Intermittent Recruitment of the Motor Units

The most remarkable result obtained from the proposed NMS model is shown in Figure 4. The intermittent recruitment of MG MUs is a phenomenon recently observed in human experiments [14], [16] and the postural control model reproduced this behaviour with a high degree of fidelity. The experimental study in [14] reported that MG MUs were intermittently recruited with a modal frequency of 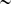
2 Hz (pooled data from 7 subjects), which is similar to the value observed in [13] for actions of a human subject manually controlling an inverted pendulum. A central hypothesis raised by several recent studies postulates the involvement of an intrinsic predictive mechanism used by the CNS in the performance of postural control [13], [15], [50]. This intrinsic mechanism, sometimes named as a “refractory response planner” [51] and involving a “psychological refractory period” [15], [50], [51], would be responsible for the intermittent actions of the neural controller during the equilibrium maintenance of an unstable load. The simulation results showed that even in the absence of any predictive mechanism (or an internal time setting neural circuit), actions of the neuronal controller occurred at a mean (modal) rate of 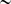
2 (4) Hz, i.e., MUs from the MG muscle were recruited with a mean (modal) interval of 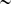
500 (250) ms, irrespective of model structure (i.e. *Model 1* and *Model 2*). The models adopted in this study are interpreted as representing a single subject instead of a population of subjects, and the MU intermittence rates observed in the simulations are within the experimental range (2–4 Hz) reported elsewhere (e.g., [11], [13], [14]). These data suggests that the interplay between a SLC and the muscles involved in the task being performed is sufficient to provide a mechanism underlying the intermittent actions of the CNS during postural control. No complex central mechanism (e.g., predictive, response planner) was needed in our model for the genesis of this control pattern.

As discussed in [14], [16], the MG muscle seems to be mostly involved in balance control during standing, while the SO muscle provides a basal torque due to its mostly continuous activity (see Figure 5 and Figure 1C). Regarding the LG muscle, a recent finding showed that this muscle has a minimal or absent activation during the postural control task [16]. The simulation data are in agreement with these experimental results, and suggest that the differences in the organisation of the MG and SO motor nuclei might be responsible for their different actions during postural control. Additionally, the LG muscle was minimally activated during the simulations (the reason why we performed quantitative analysis only on MG MUs), although the recruited LG MUs (less than 30 per simulation) followed a pattern similar to the MG MUs. This different behaviour between the lateral and medial parts of the Gastrocnemius might be explained by a different number of MNs innervating each muscle. The LG muscle has approximately 60% less MUs than the MG muscle (see Methods), and hence for a similar effective synaptic current the number of recruited MUs with similar intrinsic characteristics would be lower for the LG muscle in comparison to the MG. In the model, the SO muscle is more homogeneous, having a high number of low-threshold and smaller twitch amplitude MUs, while MG and LG muscles have an equal proportion of low- and high-threshold MUs generating higher twitch amplitudes (and mostly briefer twitches). The lower proportion of low-threshold MUs (with a similar recruitment range - see [16]) might naturally produce the intermittent recruitment due to fluctuations in sensory feedback. The rationale is that for a similar lower sensory inflow (or effective synaptic current), a low number of MG/LG MUs are recruited, hence producing a low torque at the ankle joint. Conversely, a higher number of SO MUs are recruited producing a basal torque sufficient to counterbalance the static toppling torque. During a forward sway, any small modulation of sensory inflow is sufficient to recruit additional higher-threshold MG/LG MUs, counterbalancing the postural perturbation. As the inverted pendulum returns to a backward position, sensory inflow decreases and the recruited MUs are de-recruited (see Figure 4). For the SO, these oscillations in sensory inflow seem to be lower during postural sway (see Figure 5B). However, the analysis is quite complex since the system is operating in closed loop, so that any argument based on causality may lead to logical difficulties. In spite of this limitation, the simulation results indicate that upright standing could be controlled by means of proprioceptive sensory information feedback and a mixture of *continuous* (SO muscle) and *intermittent* (mostly MG and LG muscles) action of the CNS. Therefore, the second working hypothesis raised in the present study (that efferent actions of the CNS are continuous during postural control - see Introduction) turned out to be half true, i.e., the leg muscles are activated by a combination of both continuous and intermittent processes.

The results in Figure 6 showed that MG MUs were preferentially recruited when the body leaned forward (panel A in Figure 6) and decelerated (panel B in Figure 6), i.e., MG MUs were mostly recruited in order to counteract the toppling torque due to gravity, pushing the body to a backward position. These simulation results are similar to those obtained from human subjects, as reported in [14]. The authors of the referred study proposed that a strategy of MU recruitment instead of MU rate modulation during upright standing would be generated by the CNS due to the postural task demands. The data from the simulations in Figure 6 reinforce this view. The preference for recruitment coding would be due to the same mechanisms discussed in the previous paragraph, i.e., structural features of the MG motor nucleus and modulation of sensory information due to perturbation from a mean equilibrium position. On the other hand, recent experimental and computer simulation studies have shown that during *isometric contractions*, the TS torque control relies mainly on rate coding [52] and the variability observed in both torque and EMGs is highly dependent on the MU discharge rate variability. Therefore, the same muscle group (i.e., the TS) is probably being driven according to two different laws depending on the motor task: rate coding for isometric torque control in a very stable condition, and recruitment coding (for the MG/LG muscles) in a more challenging condition, such as erect posture. Interestingly, recent experimental data relating postural sway with isometric torque variability (at similar mean torque values) in young subjects found that they have a positive correlation [53] albeit the first is much larger in magnitude than the latter. As the isometric torque control (seated subjects) involved almost certainly only continuous feedback (mostly from the SO) this experimental result gives support to the dual control mode (continuous and intermittent) that was found in the present simulations for standing posture control.

### The Role of Reciprocal Inhibition

Two model structures were simulated in order to investigate whether postural control may be influenced by the reciprocal inhibition pathway (see Methods for details). Recent studies have discussed the importance of reciprocal inhibition in movement control. For instance, [29], [30] hypothesised that this inhibitory pathway may be a better source of feedback control since TA proprioceptive activity is unmodulated by the homonymous muscle activation during postural sway.

An interesting result was that in comparison to the model without reciprocal inhibition (*Model 1*) the complete model (*Model 2*) showed an increased number of intervals in which SO muscle fibre length was negatively correlated with the COM displacement (see Figure 8). This “paradoxical” behaviour was reported in some experimental studies [29]–[31] and was used as evidence to postulate the significant role of reciprocal inhibition in the control of upright quiet standing [29]. The simulation results corroborate the hypothesis that the “paradoxical” behaviour of muscle fibre lengths may be generated by the reciprocal inhibition pathway. Nevertheless, no interval with negative correlation was found between MG and LG muscle fibre lengths and COM. In [29], the authors reported that two out of eight subjects showed a larger number of positively correlated intervals for the MG muscle, and they discussed that these subjects oscillated in a more forward position. For the physiologically-constrained set of parameters adopted in the present model the mean equilibrium position was 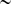
5 deg forward, which is similar to experimental findings [36]. Therefore, further studies are necessary to better understand the real significance of “paradoxical” muscle fibre behaviour and how it emerges during upright stance control. Yet, it is interesting that a highly complex and unexpected biological phenomenon may be partly explained/reproduced by a biologically plausible NMS model, and, therefore, providing neurophysiological clues to its genesis.

Regarding basic postural sway metrics (e.g., COP RMS, MV, and spectral contents) the simulation results did not show large differences between the two model structures (see Table 1), suggesting that reciprocal inhibition is not a fundamental mechanism for postural control.

In spite of the suggestion that TA muscle spindles must be a better (“cleaner”) source of ankle angle feedback than TS muscle spindles [29] the simulation results from *Model 1* (without reciprocal inhibition) showed that even “noisy” sensory feedback from the TS muscle receptors is sufficient for an adequate postural control. The TS spindle feedback is “noisy” in the sense that the TS muscle receptors are signalling a mixture of information from ankle angle changes as well as changes in muscle length and tension due to the MN pool activation.

### Model Limitations and Future Research

One conclusion that can be reached from the present simulation results is that mechanisms beyond those included in the model are not strictly necessary to reproduce experimental data from other studies. However, it is not possible to exclude that, despite theoretically not necessary, such mechanisms play a role in human postural control. Specifically, contributions from additional sensory modalities, such as foot soles, joint and skin receptors, vision, and vestibular system, certainly contribute by varying degrees to postural control depending on the particular experimental conditions [2], [3], [42], [54]. Additionally, one cannot rule out the involvement of supraspinal centres (e.g., brainstem, basal ganglia, primary motor cortex) [51], [55], specially if the maintenance of upright standing is being learned, such as in infants and adults recovering from a serious medical/neurological disease. Modulations of fusimotor [44], [56] and presynaptic inhibition activities [57], [58] are examples of important spinal-related mechanisms that certainly play relevant roles too.

In a general context, proprioceptive information from the legs is provided by muscle, joint and cutaneous receptors [54], [59]. However, in the NMS model presented here the proprioceptive information was provided exclusively by muscle receptors (i.e. muscle spindles and GTOs), which are postulated as being primary sources of sensory information in response to limb movement [27], [28]. The degree of influence of joint and cutaneous receptors on postural control is a controversial issue in the literature. Some experimental findings showed little change in postural sway after ischemia or anaesthesia [60], [61], while others showed that stimulation of cutaneous afferents evoked postural changes during quiet standing [54]. The simulation results presented here are in accordance with the former view, but further theoretical/computational and/or experimental studies are required to investigate what is the relative contribution of additional neuronal structures for the maintenance of human upright standing.

Regarding the biomechanics of the human body, it is well known that the inverted pendulum is an approximation for the human body during quiet standing [4], [26]. Other expansions, for instance, multi-link and/or multi-dimensional (e.g., including medial-lateral oscillations) models and analyses [24] are very interesting research avenues. However, any biomechanical expansion in large-scale models such as that used in the present study should envisage an increasing number of neuronal and musculoskeletal elements, along with the complexities of their interactions.

Finally, it is noteworthy that we simulated postural control during relatively brief periods (about 30 s). Prolonged unconstrained standing is associated with large changes in body equilibrium position along time [62]. A NMS model to provide approximate postural control during prolonged standing would probably require a reasonably higher complexity than the one employed in the present model and this is certainly an interesting challenge for future research.

### Concluding Remarks

Large-scale modelling has been adopted in several fields of modern neuroscience research (e.g., [32], [33], [63]–[67]). To our knowledge this is the first study aimed at modelling the NMS system involved in the control of human upright standing from a more neurophysiological perspective. The main conclusion drawn from the simulation results is that posture control might be, at least in part, mediated by spinal mechanisms, with proprioceptive information being fed back to the spinal neuronal circuitry. Additionally, the results provided evidence that complex phenomena observed in human experiments, such as intermittent actions of the CNS, might not depend on intricate control strategies of complex structures within the CNS. The structure and organisation of the spinal cord, i.e., the types of MUs, synaptic connectivities, the ordered recruitment of MUs, as well as the modulation of proprioceptive information could be sufficient to explain how the CNS controls body position during upright quiet standing in a general sense.

## Methods

### Mathematical Models

**Figure 9:**
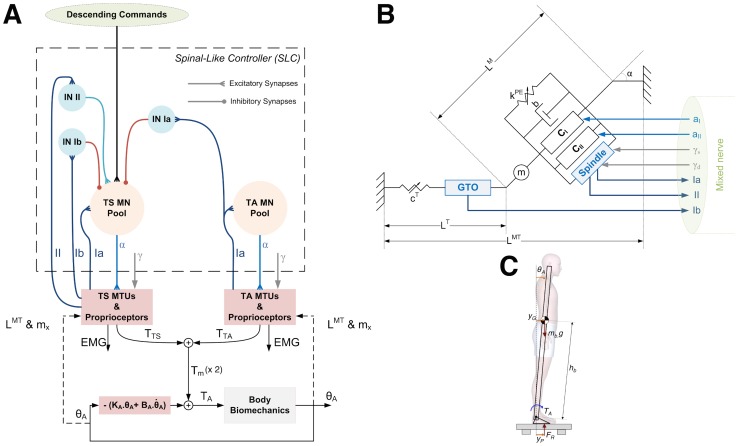
Overview of the postural control model. (A) Schematic view of the Spinal-Like Controller (SLC) and the biomechanics of the human upright standing. Mathematical models of spinal 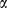 motor neurons (MNs) and interneurons (INs) make up the motor nuclei associated with the Triceps Surae (TS) and Tibialis Anterior (TA) muscles. MNs from the TS motor nuclei receive constant intensity descending commands during the maintenance of upright stance. Proprioceptive feedback is provided by Ia, II and Ib afferents from muscle spindles and Golgi tendon organs (GTOs). The information carried through these afferents is fed back to the spinal cord representing fundamental pathways (or neuronal circuits) involved in the postural control task, such as Ia monosynaptic excitation, Ib di-synaptic inhibition, group II di-synaptic excitation, and reciprocal inhibition from antagonistic Ia afferents. Activity from Gamma motor neurons (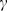-MNs) set the sensitivities of the muscle spindles. Ankle joint torque (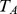) that drives the body biomechanics (to compensate for the gravitational toppling torque) is given by the sum of the torques produced by the muscles (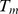) and the passive ankle joint torque, represented by the passive ankle joint stiffness (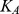) and viscosity (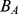). The body angle (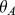) is the resultant output of the inverted pendulum acted on by gravity and by 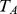. It indirectly (by means of Equations 8 and 9) defines moment arms (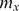) and musculotendon (MTU) lengths (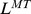), which are used to define the dynamics of both MTUs and muscle receptors (see dashed lines). Additionally, 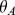 is fed back without delay to account for the intrinsic passive joint impedance (stiffness and viscosity). (B) Hill-type model used to represent the viscoelastic and contractile properties of the MTUs. Muscle fibres are represented by parallel passive elements (muscle fibre stiffness, 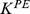, and viscosity, 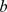) and two contractile elements (CE) representing the contractile properties of type-I and type-II muscle fibres. A pinnation angle (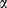) is adopted to represent the angle between the muscle fibres and the aponeurosis. In addition, a mass (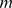) is used to increase the computational stability. Passive properties of tendon and distal aponeurosis are represented by a lumped non-linear stiffness (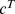). Muscle spindle is placed parallel to the muscle fibres, while the GTO is in series with the tendon. (C) Inverted pendulum model used to represent the body biomechanics during the upright quiet stance. Arrows indicate the positive direction of each variable (see the description of each variable in the text).

The model proposed in this study encompasses four main subsystems (neuronal controller, muscles, proprioceptors, and body biomechanics) that were interconnected to represent the NMS system involved in the control of human upright stance. An overview of this large-scale model is depicted in Figure 9. It is worth mentioning that the model is aimed to study body sway in the sagittal plane during unperturbed stance. In this condition, the posture control task relies mainly on afferent and efferent activities associated with the muscles around the ankle joint (ankle strategy) [9]. Figure 9A shows a schematic view of the neuronal circuitry composed of groups of spinal MNs and INs (see mathematical description below), referred to as the SLC (Spinal-Like Controller). MNs were assembled in motor nuclei associated to the TS (i.e., SO, MG, and LG) and TA muscles, which is the most relevant antagonist group of muscles involved in postural control during ankle strategy [9], [29], [36]. Stochastic descending commands represented part of the synaptic inputs from the brain that drive the spinal MNs during the maintenance of upright standing. Musculotendon units (MTUs) were represented by Hill-type models (see mathematical description below), which were driven by the spike trains from the spinal MNs (Figure 9B). Muscle spindles were placed in parallel with the muscle fibres and received commands from Gamma motor neurons (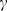-MNs), while GTOs were placed in series with the tendon. Proprioceptive feedback was provided by Ia, II and Ib axons mediating: i) monosynaptic Ia excitation; ii) di-synaptic Ib inhibition; iii) di-synaptic II excitation; and iv) reciprocal inhibition from antagonistic Ia afferents. These are fundamental pathways frequently associated with different motor tasks, including upright standing [58]. An inverted pendulum was adopted to represent the body biomechanics (Figure 9C), which is a first approximation for unperturbed quiet standing [4], [5], [10], [11], [20], [25], [26]. In the following sections, the mathematical details concerning each of these models will be provided.

#### Spinal neuron models

The MN pool model has been extensively described elsewhere [41], [52], [68], [69]. Briefly, ach type-specified MN (i.e., S-, FR-, and FF-type) was modelled as a two-compartment conductance-based neuron model. Geometrical and electrotonical parameters followed those reported in the literature for lumbar MNs of anaesthetised cats. The somatic compartment included ionic conductances responsible for the genesis of action potentials (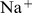 and fast 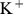) and afterhyperpolarization (slow 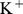). Voltage-gated ionic channels were not included in the dendritic compartment (passive-dendrite model) because they are mainly involved in the generation of persistent inward currents (PICs) and the bistable behaviour of MNs [68], [69]. It is out of the scope of this study to evaluate the possible role of PICs on human postural control. Motor axons were represented as simple spike conductors, i.e., if a MN generates a spike it is transmitted to the motor end-plate with a given delay depending on the axon conduction velocity (S-type: 44–51 m/s; FR-type: 51–52 m/s; FF-type: 52–53 m/s) and the distance between the spinal cord and the muscle (0.80 m). IN models were represented as point neurons (single-compartment) with the same ionic channels adopted in the MN models [41]. IN passive properties were based on the estimation reported in [70]. For simplicity, all IN models were supposed to have the same dynamical behaviour irrespective of the group they belong (i.e., Ia, II and Ib INs). However, spike thresholds varied linearly along the IN pool (from 10 mV to 20 mV) in order to represent the scattered recruitment of these cells within a given group. The time course of each conductance included in both MN and IN models was simplified [71] in order to speed up the simulation of thousands of neuronal elements. The models have been previously validated with respect to their biophysical properties, for instance, frequency-to-current (*f-I*) curve, afterhyperpolarization time course, and response to synaptic inputs [41], [52], [69]. Synaptic conductances followed the kinetic model proposed by [72] with parameters adjusted so that amplitude and duration of excitatory and inhibitory post-synaptic potentials matched the values reported in animal experiments (see [68], [69] for details).

As mentioned earlier, MNs were divided in four motor nuclei depending on the muscle they command. Table 2 shows the number and types of MNs adopted for each motor nucleus. These values and proportions follow estimates from the literature [73], [74] Since there is a lack of information regarding the exact number of spinal INs mediating each pathway represented in the present model, a fixed number of 350 INs was adopted for each group.

**Table 2:** Number and types of spinal motor neurons (MNs) adopted for each motor nuclei represented in the model.

Motor nuclei	S-type	FR-type	FF-type
Soleus (SO)	800	50	50
Medial Gastrocnemius (MG)	300	150	150
Lateral Gastrocnemius (LG)	130	65	65
Tibialis Anterior (TA)	250	50	50

These values were based on estimates from the literature [73], [74].

#### Model of the musculotendon units

MTUs produce both muscle torque and the surface electromyogram (EMG) (see Figure 9A). The phenomenological model of muscle EMG was extensively reported and validated in other studies [41], [52], [68]. Biomechanical properties of the muscles were represented by Hill-type models [75]–[77] containing elements as depicted in Figure 9B. A non-linear in-series elastic element (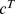) accounted for the passive property of tendon and distal aponeurosis. Two passive parallel elements (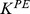 and 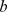) were adopted so as to represent the viscoelastic properties of the muscle fibres. In addition, the contractile properties of type-I (slow) and type-II (fast) muscle fibres were represented by two contractile elements (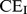 and 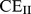), which produce active force in response to MN activation. The pinnation angle between the muscle fibres and tendon (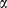) was also represented in the model. A mass (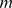) was introduced to provide computational stability. The Supplemental Material shows the frequency response of the SO muscle model that was estimated according to the experimental procedures reported in [78]. The results presented in this Supplemental are useful as a complementary validation for the modelling described in what follows.

*Activation dynamics.* Activation signals to the muscles were obtained by a filtering process (second-order critically-damped linear system) applied on the MN spike trains. The filter output was followed by a non-linear function that provided a smooth saturation [52], [79], [80]. The non-linear function (Equation (1)) accounted for the mechanisms responsible for the muscle force saturation (e.g. 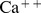 release saturation in the sarcoplasmic reticulum):
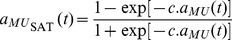
(1)in which, 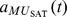 is the saturated activation signal produced by a given MU; 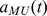 is the activation signal of a given MU before the saturation (i.e., after the second-order linear filter); and 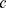 is the shape parameter of the saturation function.

For each MU the parameter 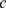 was adjusted so that the tetanic activation was achieved at a given firing rate (see [52]), i.e., a low (high) threshold MU achieved its tetanic activation at a low (high) firing rate. There was also an amplitude scaling depending on the MU type, i.e., S-type MU produced activation signals with amplitudes relatively lower than F-type MU. For more details regarding the distribution of parameter values, see [52].

The sum of all activation signals produced by S-type MUs resulted in the activation signal to the 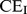 [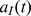]. Similarly, the activation signal to the 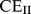 [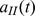] was generated by the sum of all activation signals produced by FR- and FF-type MUs. These global activation signals were normalised with respect to the maximum muscle activation, which was calculated as the sum of all tetanic activations produced by the MUs of a given muscle. A similar approach was adopted in [75].

*Passive properties.* Force-length relationships for the elastic elements were adopted from [76], [77], while the viscous force was simply proportional to the muscle fibre stretch velocity. The total parallel passive force (Equation (2)) was normalised by the maximum muscle force (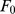) estimated from the physiological cross-sectional area of each muscle [75]. Muscle fibre length was normalised by the optimal fibre length (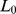). For the tendon, the adopted passive force-length relationship (Equation (3)) was the one reported by [75]. Similarly, the in-series passive force was normalised by 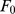 and the tendon length was normalised by 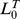, which is the value at which the force produced by the tendon equals 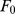.
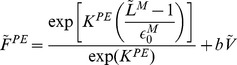
(2)in which, 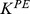 and 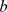 are the elastic (in 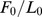) and viscous (in 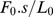) parameters, respectively; 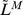 is the normalised muscle fibre length (in 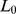); 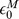 is the normalised muscle strain; and 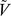 is the normalised muscle fibre velocity (in 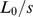).
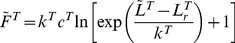
(3)in which, 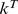 is a constant that defines the curvature of the toe region in the force-length relationship; 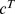 is the tendon stiffness (in 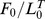); 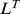 is the normalised tendon length (in 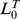); and 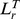 is the length at the onset of the linear region.

Muscle fibre length and tendon length are related by Equation (4). The pinnation angle varied with muscle fibre length (Equation (5)) so as to maintain the muscle thickness constant [81].
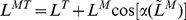
(4)in which, 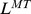 is the MTU length (in m), whose value is given by inverse kinematics data (see below); and 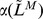 is the pinnation angle as a function of the normalised muscle fibre length.
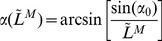
(5)in which, 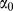 is the initial pinnation angle (in rad).

Values for the passive parameters of each muscle are presented in Table 3. These values were based on several data reported in the literature [75]–[77], [82].

**Table 3:** Passive parameters of the musculotendon units (MTUs).

Parameter (Unit)	SO	MG	LG	TA
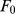 (N)	3586	1306	606	674
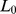 (cm)	4.90	5.70	6.40	6.80
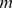 (kg)	0.53	0.22	0.12	0.15
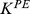 ( 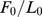 )	5			
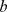 ( 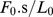 )	0.005			
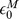 (−)	0.50			
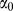 (deg)	28.30	9.90	12	9.60
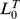 (cm)	28.90	42.40	41.30	24.90
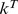 (−)	0.005			
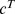 ( 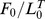 )	27.80			
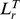 ( 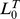 )	0.96			

These values were based on data from several studies [75]–[77], [82].

*Contraction dynamics.* The force generated by contractile elements is given by Equation (6). Force-length (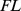) and force-velocity (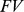) relations followed the proposal by [75] for type-I and type-II muscle fibres (see their Table 1 for details).
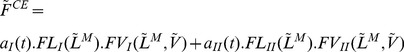
(6)


Muscle fibre velocity was calculated by integrating muscle fibre acceleration (Equation (7)), which was obtained by applying the Newton laws to the mechanical system described above (see Figure 9B for a schematic view). It is worth noting that positive velocity means muscle fibre stretch. Accordingly, muscle fibre length was calculated by integrating muscle fibre velocity.
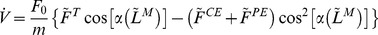
(7)


*Musculoskeletal geometry.* MTU lengths (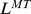) and moment arms with respect to the ankle joint (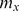) were calculated according to the approach proposed by [83]. For each muscle, fourth-order polynomials were used to adjust the relations between MTU length and ankle joint (Equation (8)), as well as moment arm (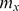) and ankle joint angle (Equation (9)). The coefficients of these functions are presented in Table 4. Relations for the MG and LG (biarticular muscles) were obtained for the knee angle at zero degree (fully extended). Inverse kinematic data were reported in [82].

**Table 4:** Coefficients of equations (8) and (9).

Parameter (Unit)	SO	MG	LG	TA
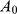 (cm)	32.30	46.40	45.50	30.60
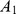 (cm/deg)	7.22 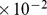	7.48 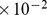	7.62 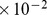	−7.44 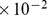
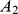 (cm/ 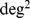 )	−2.24 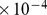	−1.13 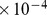	−1.25 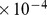	−1.41 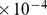
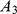 (cm/ 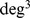 )	−3.15 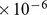	−3.50 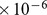	−3.55 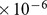	2.42 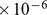
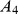 (cm/ 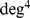 )	9.27 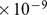	7.35 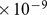	7.65 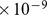	1.50 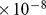
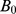 (cm)	−4.10	−4.30	−4.40	4.30
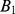 (cm/deg)	2.57 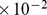	1.30 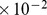	1.44 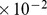	1.66 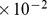
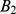 (cm/ 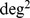 )	5.45 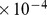	6.08 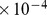	6.18 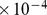	−3.89 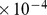
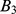 (cm/ 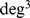 )	−2.22 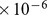	−1.87 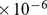	−1.94 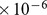	−4.45 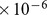
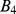 (cm/ 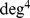 )	−5.50 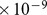	−1.02 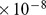	−1.02 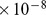	−4.34 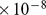

These values were estimated from inverse kinematics data (least-squares curve fitting) reported in [82].



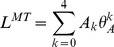
(8)

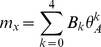
(9)


#### Models of proprioceptors and afferent fibres

A muscle spindle model previously reported by [84] was placed parallel to the muscle fibres (see Figure 9B). It represents the dynamics of bag 1, bag 2 and chain intrafusal muscle fibres. Ia mean firing rate is a non-linear function of all three instrafusal fibre outputs, hence conveying both static and dynamic information about muscle stretch. On the other hand, type II mean firing rate is produced by a contribution of bag 2 and chain intrafusal fibre outputs, which are dependent on the static component of muscle fibre stretch [84]. Normalised muscle fibre length (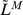) is the input to the muscle spindle model. In addition, bag 1 intrafusal fibre is sensitive to dynamic fusimotor activity (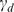), while bag 2 and chain intrafusal fibres are sensitive to static fusimotor activity (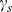). In this study, biophysical properties of 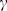-MNs were not represented. Static and dynamic fusimotor activities were both modelled as Gaussian random processes with variance equal to 3% of the mean value (empirically chosen). The mean value was adjusted in order to stabilize the biomechanical system (see Simulation Protocol below). Almost all model parameters were maintained equal to those reported by [84] (see their Table 1); however, the gain of each intrafusal fibre was adjusted (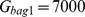, 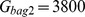, and 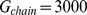) in order to produce Ia and type II firing rates compatible with experimental data from humans [59].

A GTO model reported by [85] was in-series with the tendon (see Figure 9B). It represents the lumped dynamics of a population of Ib afferents in response to the force produced by the tendon (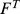). Basically, this model encompasses a static non-linear function (Equation (10)) in series with a linear dynamics (Equation (11)) resulting in the afferent firing rate [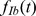]. The transfer function that represents the linear dynamics was transformed to a digital filter (bilinear transformation) so as to reduce the computational complexity of the system. Individual Ib afferent activity was obtained by dividing the GTO model output by the total number of Ib afferents in each muscle. Model parameters were chosen to produce individual Ib firing rates compatible with those reported in the literature [59].
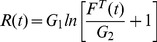
(10)in which, 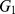 and 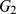 are adjustable parameters, chosen equal to 60 Hz and 4 N, respectively.
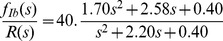
(11)in which, 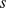 is the Laplace variable; 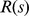 is the Laplace transform of 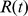 (Equation (10)); and 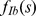 is the Laplace transform of the global Ib firing rate.

The outputs of the modelled muscle receptors are continuous functions of time that represent the instantaneous firing rate of Ia, II, and Ib afferents. These continuous functions [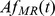] (with 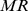 being either, Ia, II, or Ib muscle receptor afferent) were converted into independent stochastic point processes to represent the spiking activity travelling along a bundle of afferent fibres with a given conduction velocity. The 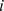-th spike train was modelled as a non-homogeneous Gamma point process with a mean intensity [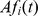] modulated by the output of a muscle receptor (spindle or GTO) and with an order (or shape factor) adjusted to produce a variability compatible with experimental data [86]. The total number of afferents for each muscle (see Table 5) and conduction velocities (see Table 6) were based on estimates from the literature [58], [87], [88]. Afferent fibres were recruited according to a recruitment law given by Equation 12 [79].

**Table 5:** Number and types of afferent fibres adopted for each muscle represented in the model.

Muscle	Ia	II	Ib
Soleus (SO)	400	500	300
Medial Gastrocnemius (MG)	160	200	120
Lateral Gastrocnemius (LG)	160	200	120
Tibialis Anterior (TA)	280	350	140

These values were based on estimates from the literature [58], [88].

**Table 6:** Sensory feedback parameters adopted in the model.

Parameter	Value	Unit
Conduction velocity: Ia afferents†	62–67	m/s
Conduction velocity: Ib afferents†	56–62	m/s
Conduction velocity: type II afferents†	30–35	m/s
Connectivity: Ia afferents to MNs (homonymous muscle)	80	%
Connectivity: Ia afferents to MNs (synergist muscle)‡	15	%
Connectivity: Ia afferents to INs	70	%
Connectivity: Ib and type II afferents to INs	30	%
Connectivity: Ia inhibitory INs to MNs	15	%
Connectivity: Ib inhibitory INs to MNs	10	%
Connectivity: group II excitatory INs to MNs	20	%
Maximum conductance: Ia afferents to MNs	600	nS
Maximum conductance: Ia afferents to INs	600	nS
Maximum conductance: type II afferents to INs	450	nS
Maximum conductance: Ib afferents to INs	300	nS
Maximum conductance: INs to MNs	300	nS

† These values varied linearly along the bundle of sensory afferents.

‡ There is no connection between Ia afferents from MG and SO MNs [58].



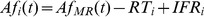
(12)in which, 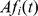 is the mean firing rate (intensity) of the 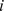-th afferent; 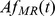 is the output of the muscle receptor models (spindle or GTO) for a given afferent type; 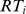 and 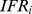 are the recruitment threshold and the initial firing rate of the 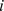-th afferent, respectively.

The 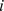-th afferent was recruited when the output produced by a specific muscle receptor afferent [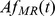] crossed a recruitment threshold (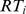). Irrespective of the afferent type (i.e., Ia, II, and Ib) the 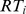 was linearly varied along the afferents from 0–50 Hz, i.e., when the specific muscle receptor output reached 50 Hz all afferent fibres of a given type were recruited. At the time of recruitment the 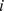-th afferent fibre discharged with an initial firing rate (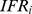) that was randomly varied along the bundle of afferents following a Gaussian random number (mean  = 5 Hz; standard-deviation  = 2.50 Hz). The values of 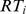 and 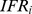 were empirically chosen so as to produce the recruitment behaviour of afferent fibres observed in human experiments [59], [89]. Table 6 shows the connectivity ratios between sensory afferents and the spinal neurons (MNs and INs), with values based on data reported in [58]. Maximum synaptic conductances (see Table 6) were empirically adjusted in order to produce a stable and meaningful model response.

#### Body biomechanics

As mentioned above, body biomechanics during the upright quiet stance was represented by an inverted pendulum (Figure 9C). Equations of motion of body COM with respect to the ankle joint (fixed pivot point) are given by Equations (13) and (14). It is noteworthy that our analysis was restricted to the sagittal plane, which represents the anteroposterior displacement of the body COM. Anthropometric parameters were empirically chosen to represent a young adult with 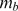  = 60 kg and 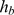  = 0.85 m.
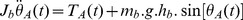
(13)in which, 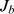 is the body inertia (in kg.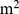) given by 
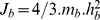
; 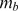 is the body mass excluding the feet (in kg); 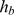 is the COM height with respect to the ankle joint (in m); 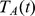 is the restoring torque around the ankle joint (see Equation (13)); and 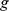 is the gravity (set to 9.81 m/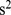).
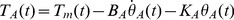
(14)in which, 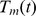 is torque produced by the leg muscles (in Nm); 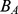 and 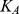 are the viscoelastic coefficients that represent the passive property of the joint (in Nm.s/rad and Nm/rad, respectively).

Muscular torque (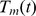) is given by the net torque produced by the TS and the TA muscles multiplied by two (for simplicity, we have assumed that both legs produce the same torque and have the same control mechanism). The torque produced by the TA muscle was defined as positive, so that activation of the TS tends to restore the body from the toppling torque produced by gravity. The viscoelastic passive torque was introduced in the model because Hill-type muscle models cannot properly produce muscle stiffness [47]. A preliminary simulation was performed in order to estimate the ankle joint stiffness due to the passive properties of the modelled Hill-type MTUs. In this simulation the neuronal controller was turned off and the ankle joint angle [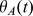] was randomly varied (Gaussian stochastic process). The resultant (passive) muscle torque [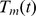] was measured at the output of the model. A spectral analysis was carried out to estimate the resultant passive ankle joint impedance (see [90], [91]), and the passive ankle joint stiffness due to the Hill-type MTUs was estimated as the mean impedance value in the range 0–1 Hz (static component). The passive ankle joint stiffness produced by the Hill-type MTUs was estimated to be only about 5% of the critical stiffness (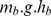). An additional passive ankle stiffness (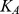) was adopted equal to 65% 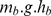, resulting in a total passive ankle joint stiffness of about 70% [46], [48]. A small viscosity (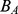 equal to 5.81 Nm.s/rad [46]) was also included in the model. These viscoelastic intrinsic properties of the ankle joint accounted for part of the postural control through a mechanism frequently referred to as *preflexes* [92] (see Figure 9A).

*COM and COP displacements.* Typically in human experiments, both COM (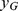) and COP (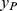) displacements (projected on the anteroposterior axis in the present study) have been recorded to evaluate the body sway during upright stance. Here, these biomechanical variables were calculated according to Equations (15) and (16).
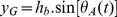
(15)

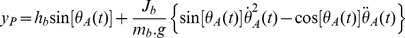
(16)


### Simulation Protocol

The models described above were written in Java programming language (Oracle Corp) and simulated in an open-source web-based application developed in the Eclipse IDE (The Eclipse Foundation). Model source code is available for download at the website http://remoto.leb.usp.br/ and at a public repository (http://dx.doi.org/10.6084/m9.figshare.1029085). Differential equations were numerically solved by a fourth-order Runge-Kutta method with a 50* µs* time step.

Recent experimental studies argued that the reciprocal inhibition from antagonistic Ia afferents (from TA muscle spindles to TS MNs - see Figure 9 A) has an important role in the postural control due to the “orthodox” modulation of TA length and the lack of TA contraction during standing [16], [29]. To investigate this potential role of reciprocal inhibition pathway, two different model structures were used in this study. The first one, hereafter referred to as *Model 1*, excluded the reciprocal inhibition pathway so that all proprioceptive information was provided exclusively by the TS sensory afferents (autogenic pathway). Conversely, *Model 2* is the complete model fully described above. All common parts in both models have the same parameter values.

Three independent simulations (30-s duration) were carried out with each model. During the first second of simulation, the movement of the inverted pendulum was restricted so that the neuromuscular system reached its steady-state condition. This prevented instabilities due to the lack of neural activity. In order to provide a basal activation, descending commands were modelled as 400 homogeneous stochastic Gamma point processes with an individual mean rate equal to 50 Hz and a shape factor equal to 25 (coefficient of variation equal to 20%). With this descending drive (without any feedback information) the MN pools produced a small basal muscle torque equal to approximately 2% of the maximum torque. As mentioned earlier, the fusimotor drive was modelled as a Gaussian stochastic process with a small variance. The mean values of static and dynamic fusimotor activities were adjusted for each simulation so as to produce outputs in which the inverted pendulum was oscillating around an equilibrium point for the whole simulation duration. For *Model 1* the values of static (dynamic) fusimotor activities were 31.10 (33.30), 31.20 (33.30), and 31.50 (33.60). Similarly, for *Model 2* the values of static (dynamic) fusimotor activities were set to 32 (33.80), 32 (33), and 31.70 (32.70).

### Data Analysis

Several output variables were analysed in the present study: i) Spike trains from MNs, INs and afferent fibres; ii) EMGs from the TS muscles; iii) COM and COP anteroposterior displacements; iv) Muscular torque; v) Muscle fibre lengths; vi) Joint angle and angular velocity. These raw variables are available for download at a public repository (Model 1 - http://dx.doi.org/10.6084/m9.figshare.1027609; Model 2 - http://dx.doi.org/10.6084/m9.figshare.1029084). Analyses were performed on 22.50-s duration time series, so that the initial 5 s and final 2.50 s were removed due to transient responses and data filtering (see below).

COM, COP, joint angle, angular velocity, muscle fibre length, and torque time series were detrended and downsampled to 2 kHz. In order to compare model behaviour with experimental data recorded with force plates, time- and frequency-domain metrics were estimated from the COP, for instance, RMS, MV and 50% power frequency [42]. Power spectra were estimated by Welch's method. EMGs were downsampled to 2 kHz, rectified, and lowpass filtered at 2 Hz (zero-phase fourth-order Butterworth filter).

Similar to the studies reported in [9], [19], [43], cross-correlation analysis was performed between COM and COP time series, as well as COP and EMGs. In addition, similar to the analysis reported in [29], correlation coefficients were computed between COM and muscle fibre lengths. For the latter analysis COM and muscle fibre length time series were binned to 3-s duration intervals (without overlap) so that the correlation coefficients were calculated for each bin (see Figure 7).

In order to quantify the degree of intermittency of a given muscle, the activation ratio of individual MUs was measured. This ratio was calculated as the percentage of time a given MU had interspike intervals lower than 250 ms [16]. An activation ratio equal to 0 indicates that the MU was inactive during the whole simulation time, whereas a ratio equal to 1 indicates a continuous activity. Therefore, lower activation ratios represent an intermittently discharging MU. Additionally, phase plots were constructed to evaluate the MU recruitment during quiet standing. Recruitment phase plots were constructed by binning joint angle, angular velocity, muscular torque and spike trains in 100-ms non-overlapping windows. Within each window, the number of recruited MUs was counted and a circle with diameter proportional to the counting number was plotted in 2-dimension angle-velocity (see Figure 6A) or angle-torque graphs (see Figure 6B). Data from the three simulations were pooled together in the same phase plot. A similar analysis was performed in [14].

When applicable, statistical analysis was performed with a significance level set at 0.05. All analyses were performed in Matlab (The MathWorks Inc) and SPSS Statistics (IBM Corp).

## Supporting Information

Supporting Information S1 **Frequency response of muscle model.** This supporting information file provides additional validation data for the muscle model.
(PDF)

## References

[1] Macpherson JM, Horak FB (2013) Posture. In: Kandel ER, Schwartz JH, Jessell TM, Siegelbaum SA, Hudspeth AJ, editors, Principles of neural science, New York: McGraw-Hill, chapter 41. 5 edition, pp. 935–959.

[2] Simoneau GG, Ulbrecht JS, Derr JA, Cavanagh PR (1995) Role of somatosensory input in the control of human posture. Gait & Posture 3: 115–122.

[3] Baloh RW, Jacobson KM, Beykirch K, Honrubia V (1998) Static and dynamic posturography in patients with vestibular and cerebellar lesions. Archives of Neurology 55: 649–654. 9605721 10.1001/archneur.55.5.649

[4] Peterka RJ (2000) Postural control model interpretation of stabilogram diffusion analysis. Biological Cybernetics 82: 335–343. 10804065 10.1007/s004220050587

[5] Maurer C, Peterka RJ (2005) A new interpretation of spontaneous sway measures based on a simple model of human postural control. Journal of Neurophysiology 93: 189–200. 15331614 10.1152/jn.00221.2004

[6] van der Kooij H, de Vlugt E (2007) Postural responses evoked by platform pertubations are dominated by continuous feedback. Journal of Neurophysiology 98: 730–743. 17460106 10.1152/jn.00457.2006

[7] Fitzpatrick R, Burke D, Gandevia SC (1996) Loop gain of reflexes controlling human standing measured with the use of postural and vestibular disturbances. Journal of Neurophysiology 76: 3994–4008. 8985895 10.1152/jn.1996.76.6.3994

[8] Morasso PG, Schieppati M (1999) Can muscle stiffness alone stabilize upright standing? Journal of Neurophysiology 82: 1622–1626. 10482776 10.1152/jn.1999.82.3.1622

[9] Gatev P, Thomas S, Kepple T, Hallett M (1999) Feedforward ankle strategy of balance during quiet stance in adults. The Journal of Physiology 514: 915–928. 9882761 10.1111/j.1469-7793.1999.915ad.x PMC2269093

[10] Bottaro A, Casadio M, Morasso PG, Sanguineti V (2005) Body sway during quiet standing: is it the residual chattering of an intermittent stabilization process? Human Movement Science 24: 588–615. 16143414 10.1016/j.humov.2005.07.006

[11] Bottaro A, Yasutake Y, Nomura T, Casadio M, Morasso P (2008) Bounded stability of the quiet standing posture: an intermittent control model. Human Movement Science 27: 473–95. 18342382 10.1016/j.humov.2007.11.005

[12] Gawthrop P, Loram I, Lakie M, Gollee H (2011) Intermittent control: a computational theory of human control. Biological Cybernetics 104: 31–51. 21327829 10.1007/s00422-010-0416-4

[13] Loram ID, Gollee H, Lakie M, Gawthrop PJ (2011) Human control of an inverted pendulum: is continuous control necessary? Is intermittent control effective? Is intermittent control physiological? Journal of Physiology 589: 307–324. 21098004 10.1113/jphysiol.2010.194712 PMC3043535

[14] Vieira TM, Loram ID, Muceli S, Merletti R, Farina D (2012) Recruitment of motor units in the medial gastrocnemius muscle during human quiet standing: is recruitment intermittent? What triggers recruitment? Journal of Neurophysiology 107: 666–676. 21994258 10.1152/jn.00659.2011 PMC3349625

[15] van de Kamp C, Gawthrop PJ, Gollee H, Loram ID (2013) Refractoriness in sustained visuo-manual control: is the refractory duration intrinsic or does it depend on external system properties? PLoS Computational Biology 9: e1002843. 23300430 10.1371/journal.pcbi.1002843 PMC3536613

[16] Héroux ME, Dakin CJ, Luu BL, Inglis JT, Blouin JS (2014) Absence of lateral gastrocnemius activity and differential motor unit behavior in soleus and medial gastrocnemius during standing balance. Journal of Applied Physiology 116: 140–8. 24311748 10.1152/japplphysiol.00906.2013 PMC3921363

[17] Cabrera J, Milton J (2002) On-Off intermittency in a human balancing task. Physical Review Letters 89: 158702. 12366030 10.1103/PhysRevLett.89.158702

[18] Alexandrov AV, Frolov AA, Horak FB, Carlson-Kuhta P, Park S (2005) Feedback equilibrium control during human standing. Biological Cybernetics 93: 309–322. 16228222 10.1007/s00422-005-0004-1 PMC1430400

[19] Kohn AF (2005) Cross-correlation between EMG and center of gravity during quiet stance: theory and simulations. Biological Cybernetics 93: 382–388. 16189672 10.1007/s00422-005-0016-x

[20] Masani K, Vette AH, Popovic MR (2006) Controlling balance during quiet standing: proportional and derivative controller generates preceding motor command to body sway position observed in experiments. Gait & Posture 23: 164–172. 16399512 10.1016/j.gaitpost.2005.01.006

[21] Kuo AD (2005) An optimal state estimation model of sensory integration in human postural balance. Journal of Neural Engineering 2: S235–49. 16135887 10.1088/1741-2560/2/3/S07

[22] van der Kooij H, Jacobs R, Koopman B, Grootenboer H (1999) A multisensory integration model of human stance control. Biological Cybernetics 80: 299–308. 10365423 10.1007/s004220050527

[23] van der Kooij H, Jacobs R, Koopman B, Van der Helm F (2001) An adaptive model of sensory integration in a dynamic environment applied to human stance control. Biological Cybernetics 84: 103–115. 11205347 10.1007/s004220000196

[24] Suzuki Y, Nomura T, Casadio M, Morasso P (2012) Intermittent control with ankle, hip, and mixed strategies during quiet standing: a theoretical proposal based on a double inverted pendulum model. Journal of Theoretical Biology 310: 55–79. 22732276 10.1016/j.jtbi.2012.06.019

[25] Nomura T, Oshikawa S, Suzuki Y, Kiyono K, Morasso P (2013) Modeling human postural sway using an intermittent control and hemodynamic perturbations. Mathematical Biosciences 245: 86–95. 23435118 10.1016/j.mbs.2013.02.002

[26] Gage WH, Winter DA, Frank JS, Adkin AL (2004) Kinematic and kinetic validity of the inverted pendulum model in quiet standing. Gait & Posture 19: 124–132. 15013500 10.1016/S0966-6362(03)00037-7

[27] Scott SH, Loeb GE (1994) The computation of position sense from spindles in monoarticular and multiarticular muscles. Journal of Neuroscience 14: 7529–7540. 7996193 10.1523/JNEUROSCI.14-12-07529.1994 PMC6576884

[28] Proske U, Wise AK, Gregory JE (2000) The role of muscle receptors in the detection of movements. Progress in Neurobiology 60: 85–96. 10622377 10.1016/s0301-0082(99)00022-2

[29] Di Giulio I, Maganaris CN, Baltzopoulos V, Loram ID (2009) The proprioceptive and agonist roles of gastrocnemius, soleus and tibialis anterior muscles in maintaining human upright posture. The Journal of Physiology 587: 2399–2416. 19289550 10.1113/jphysiol.2009.168690 PMC2697307

[30] Day JT, Lichtwark GA, Cresswell AG (2013) Tibialis anterior muscle fascicle dynamics adequately represent postural sway during standing balance. Journal of Applied Physiology 115: 1742–50. 24136108 10.1152/japplphysiol.00517.2013

[31] Loram ID, Maganaris CN, Lakie M (2005) Active, non-spring-like muscle movements in human postural sway: how might paradoxical changes in muscle length be produced? Journal of Physiology 564: 281–293. 15661825 10.1113/jphysiol.2004.073437 PMC1456051

[32] Schuurmans J, van der Helm FC, Schouten AC (2010) Relating reflex gain modulation in posture control to underlying neural network properties using a neuromusculoskeletal model. Journal of Computational Neuroscience 30: 555–65.. 20865310 10.1007/s10827-010-0278-8 PMC3108017

[33] Stienen AHA, Schouten AC, Schuurmans J, van der Helm FCT (2007) Analysis of reflex modulation with a biologically realistic neural network. Journal of Computational Neuroscience 23: 333–348. 17503169 10.1007/s10827-007-0037-7 PMC2799624

[34] Elias LA, Watanabe RN, Kohn AF (2013) Large-scale neuromusculoskeletal model used to investigate neurophysiological mechanisms behind upright stance control. In: 43rd Annual Meeting of the Society for Neuroscience. San Diego, p. 293.07.

[35] Elias LA, Watanabe RN, Kohn AF (2014) The role of reciprocal inhibition on human postural control. In: Proceedings of the Bernstein Conference 2014. Göttingen: BFNT/BCCN, pp. 185–186. doi:10.12751/nncn.bc2014.0202.

[36] Mezzarane RA, Kohn AF (2007) Control of upright stance over inclined surfaces. Experimental Brain Research 180: 377–388. 17279384 10.1007/s00221-007-0865-8

[37] Taube W, Gruber M, Beck S, Faist M, Gollhofer A, et al (2007) Cortical and spinal adaptations induced by balance training: correlation between stance stability and corticospinal activation. Acta Physiologica 189: 347–358. 17263693 10.1111/j.1748-1716.2007.01665.x

[38] Ushiyama J, Takahashi Y, Ushiba J (2010) Muscle dependency of corticomuscular coherence in upper and lower limb muscles and training-related alterations in ballet dancers and weightlifters Muscle dependency of corticomuscular coherence in upper and lower limb muscles and training-related alterat. Journal of Applied Physiology 109: 1086–95. 20689093 10.1152/japplphysiol.00869.2009

[39] Lawrence EL, Fassola I, Werner I, Leclercq C, Valero-Cuevas FJ (2014) Quantification of dexterity as the dynamical regulation of instabilities: comparisons across gender, age, and disease. Frontiers in Neurology 5: 53. 24782824 10.3389/fneur.2014.00053 PMC3995042

[40] Murnaghan CD, Squair JW, Chua R, Inglis JT, Carpenter MG (2014) Cortical contributions to control of posture during unrestricted and restricted stance. Journal of Neurophysiology 111: 1920–6. 24523526 10.1152/jn.00853.2012

[41] Cisi RRL, Kohn AF (2008) Simulation system of spinal cord motor nuclei and associated nerves and muscles, in a Web-based architecture. Journal of Computational Neuroscience 25: 520–542. 18506610 10.1007/s10827-008-0092-8

[42] Prieto TE, Myklebust JB, Hoffmann RG, Lovett EG, Myklebust BM (1996) Measures of postural steadiness: Differences between healthy young and elderly adults. IEEE Transactions on Biomedical Engineering 43: 956–966. 9214811 10.1109/10.532130

[43] Masani K, Popovic MR, Nakazawa K, Kouzaki M, Nozaki D (2003) Importance of body sway velocity information in controlling ankle extensor activities during quiet stance. Journal of Neurophysiology 90: 3774–82. 12944529 10.1152/jn.00730.2002

[44] Prochazka A, Hulliger M, Zangger P, Appenteng K (1985) ‘Fusimotor set’: new evidence for alpha-independent control of gamma-motoneurones during movement in the awake cat. Brain Research 339: 136–40. 3161585 10.1016/0006-8993(85)90632-8

[45] Windhorst U (2007) Muscle proprioceptive feedback and spinal networks. Brain Research Bulletin 73: 155–202. 17562384 10.1016/j.brainresbull.2007.03.010

[46] Casadio M, Morasso PG, Sanguineti V (2005) Direct measurement of ankle stiffness during quiet standing: implications for control modelling and clinical application. Gait & Posture 21: 410–424. 15886131 10.1016/j.gaitpost.2004.05.005

[47] Loram ID, Maganaris CN, Lakie M (2007) The passive, human calf muscles in relation to standing: the non-linear decrease from short range to long range stiffness. Journal of Physiology-London 584: 661–675. 10.1113/jphysiol.2007.140046 PMC2277155 17823209

[48] Loram ID, Maganaris CN, Lakie M (2007) The passive, human calf muscles in relation to standing: the short range stiffness lies in the contractile component. Journal of Physiology-London 584: 677–692. 10.1113/jphysiol.2007.140053 PMC2277144 17823208

[49] van Soest AJ, Rozendaal LA (2008) The inverted pendulum model of bipedal standing cannot be stabilized through direct feedback of force and contractile element length and velocity at realistic series elastic element stiffness. Biological Cybernetics 99: 29–41. 18584202 10.1007/s00422-008-0240-2

[50] van de Kamp C, Gawthrop PJ, Gollee H, Lakie M, Loram ID (2013) Interfacing sensory input with motor output: does the control architecture converge to a serial process along a single channel? Frontiers in Computational Neuroscience 7: 55. 23675342 10.3389/fncom.2013.00055 PMC3648771

[51] Loram ID, van de Kamp C, Lakie M, Gollee H, Gawthrop PJ (2014) Does the motor system need intermittent control? Exercise and Sport Sciences Reviews 42: 117–25. 24819544 10.1249/JES.0000000000000018

[52] Watanabe RN, Magalhães FH, Elias LA, Chaud VM, Mello EM, et al (2013) Influences of premotoneuronal command statistics on the scaling of motor output variability during isometric plantar flexion. Journal of Neurophysiology 110: 2592–606. 24027105 10.1152/jn.00073.2013

[53] Mello EM, Magalhães FH, Kohn AF (2013) Larger plantar flexion torque variability implies less stable balance in the young: an association affected by knee position. Human Movement Science 32: 1310–24. 24060221 10.1016/j.humov.2013.05.004

[54] Kavounoudias A, Roll R, Roll JP (2001) Foot sole and ankle muscle inputs contribute jointly to human erect posture regulation. Journal of Physiology-London 532: 869–878. 10.1111/j.1469-7793.2001.0869e.x PMC2278585 11313452

[55] Jacobs JV, Horak FB (2007) Cortical control of postural responses. Journal of Neural Transmission 114: 1339–1348. 17393068 10.1007/s00702-007-0657-0 PMC4382099

[56] Proske U, Gandevia SC (2012) The proprioceptive senses: their roles in signaling body shape, body position and movement, and muscle force. Physiological Reviews 92: 1651–97. 23073629 10.1152/physrev.00048.2011

[57] Zehr EP (2002) Considerations for use of the Hoffmann reflex in exercise studies. European Journal of Applied Physiology 86: 455–68. 11944092 10.1007/s00421-002-0577-5

[58] Pierrot-Deseilligny E, Burke D (2012) The circuitry of the human spinal cord: Spinal and corticospinal mechanisms of movement. Cambridge: Cambridge University Press, 606 pp.

[59] Aniss AM, Diener HC, Hore J, Gandevia SC, Burke D (1990) Behavior of human muscle receptors when reliant on proprioceptive feedback during standing. Journal of Neurophysiology 64: 661–670. 2213138 10.1152/jn.1990.64.2.661

[60] Diener HC, Dichgans J, Guschlbauer B, Mau H (1984) The significance of proprioception on postural stabilization as assessed by ischemia. Brain Research 296: 103–9. 6713202 10.1016/0006-8993(84)90515-8

[61] Meyer PF, Oddsson LIE, De Luca CJ (2004) The role of plantar cutaneous sensation in unperturbed stance. Experimental Brain Research 156: 505–12. 14968274 10.1007/s00221-003-1804-y

[62] Duarte M, Harvey W, Zatsiorsky VM (2000) Stabilographic analysis of unconstrained standing. Ergonomics 43: 1824–1839. 11105975 10.1080/00140130050174491

[63] Markram H (2006) The blue brain project. Nature Reviews Neuroscience 7: 153–160. 16429124 10.1038/nrn1848

[64] Raphael G, Tsianos GA, Loeb GE (2010) Spinal-like regulator facilitates control of a two-degree-of-freedom wrist. Journal of Neuroscience 30: 9431–9444. 20631172 10.1523/JNEUROSCI.5537-09.2010 PMC6632449

[65] Eliasmith C, Stewart TC, Choo X, Bekolay T, DeWolf T, et al (2012) A large-scale model of the functioning brain. Science 338: 1202–5. 23197532 10.1126/science.1225266

[66] Dideriksen JL, Negro F, Enoka RM, Farina D (2012) Motor unit recruitment strategies and muscle properties determine the influence of synaptic noise on force steadiness. Journal of Neurophysiology 107: 3357–3369. 22423000 10.1152/jn.00938.2011 PMC3378401

[67] Farina D, Negro F, Dideriksen JL (2014) The effective neural drive to muscles is the common synaptic input to motor neurons. The Journal of Physiology 592: 3427–41.. 24860172 10.1113/jphysiol.2014.273581 PMC4229341

[68] Elias LA, Chaud VM, Kohn AF (2012) Models of passive and active dendrite motoneuron pools and their differences in muscle force control. Journal of Computational Neuroscience 33: 515–531. 22562305 10.1007/s10827-012-0398-4

[69] Elias LA, Kohn AF (2013) Individual and collective properties of computationally efficient motoneuron models of types S and F with active dendrites. Neurocomputing 99: 521–533.

[70] Bui TV, Grande G, Rose PK (2008) Multiple modes of amplification of synaptic inhibition to motoneurons by persistent inward currents. Journal of Neurophysiology 99: 571–582. 18046007 10.1152/jn.00717.2007 PMC2930909

[71] Destexhe A (1997) Conductance-based integrate-and-fire models. Neural Computation 9: 503–514. 9097470 10.1162/neco.1997.9.3.503

[72] Destexhe A, Mainen ZF, Sejnowski TJ (1994) An efficient method for computing synaptic conductances based on a kinetic-model of receptor-binding. Neural Computation 6: 14–18.

[73] Johnson MA, Polgar J, Weightman D, Appleton D (1973) Data on the distribution of fibre types in thirty-six human muscles. An autopsy study. Journal of the Neurological Sciences 18: 111–129. 4120482 10.1016/0022-510x(73)90023-3

[74] McComas AJ (1991) Invited review: motor unit estimation: methods, results, and present status. Muscle & Nerve 14: 585–597. 1922165 10.1002/mus.880140702

[75] Cheng EJ, Brown IE, Loeb GE (2000) Virtual muscle: a computational approach to understanding the effects of muscle properties on motor control. Journal of Neuroscience Methods 101: 117–130. 10996372 10.1016/s0165-0270(00)00258-2

[76] Thelen DG (2003) Adjustment of muscle mechanics model parameters to simulate dynamic contractions in older adults. Journal of Biomechanical Engineering 125: 70–77. 12661198 10.1115/1.1531112

[77] de Vlugt E, de Groot JH, Wisman WH, Meskers CG (2012) Clonus is explained from increased reflex gain and enlarged tissue viscoelasticity. Journal of Biomechanics 45: 148–155. 22014329 10.1016/j.jbiomech.2011.09.023

[78] Bawa P, Stein RB (1976) Frequency-response of human soleus muscle. Journal of Neurophysiology 39: 788–793. 966038 10.1152/jn.1976.39.4.788

[79] Chaud VM, Elias LA, Watanabe RN, Kohn AF (2012) A simulation study of the effects of activation-dependent muscle stiffness on proprioceptive feedback and short-latency reflex. In: 4th IEEE RAS/EMBS International Conference on Biomedical Robotics and Biomechatronics. Rome: IEEE, pp. 133–138. doi:10.1109/BioRob.2012.6290871.

[80] Watanabe RN, Elias LA, Kohn AF (2013) Low-frequency fluctuations of plantar flexion torque in force and position control tasks studied experimentally and by a neuromusculoskeletal model. In: 6th International IEEE/EMBS Conference on Neural Engineering (NER). IEEE, pp. 794–797. doi:10.1109/NER.2013.6696054.

[81] Sartori M, Reggiani M, Farina D, Lloyd DG (2012) EMG-driven forward-dynamic estimation of muscle force and joint moment about multiple degrees of freedom in the human lower extremity. PLoS One 7: e52618. 23300725 10.1371/journal.pone.0052618 PMC3530468

[82] Arnold EM, Ward SR, Lieber RL, Delp SL (2010) A model of the lower limb for analysis of human movement. Annals of Biomedical Engineering 38: 269–279. 19957039 10.1007/s10439-009-9852-5 PMC2903973

[83] Menegaldo LL, de Toledo Fleury A, Weber HI (2004) Moment arms and musculotendon lengths estimation for a three-dimensional lower-limb model. Journal of Biomechanics 37: 1447–1453. 15275854 10.1016/j.jbiomech.2003.12.017

[84] Mileusnic MP, Brown IE, Lan N, Loeb GE (2006) Mathematical models of proprioceptors. I. Control and transduction in the muscle spindle. Journal of Neurophysiology 96: 1772–1788. 16672301 10.1152/jn.00868.2005

[85] Lin CC, Crago PE (2002) Neural and mechanical contributions to the stretch reflex: a model synthesis. Annals of Biomedical Engineering 30: 54–67. 11874142 10.1114/1.1432692

[86] Matthews PB, Stein RB (1969) Regularity of primary and secondary muscle spindle afferent discharges. Journal of Physiology-London 202: 59–82. 10.1113/jphysiol.1969.sp008795 PMC1351465 4238988

[87] Nardone A, Schieppati M (1998) Medium-latency response to muscle stretch in human lower limb: estimation of conduction velocity of group II fibres and central delay. Neuroscience Letters 249: 29–32. 9672381 10.1016/s0304-3940(98)00383-8

[88] Banks RW (2006) An allometric analysis of the number of muscle spindles in mammalian skeletal muscles. Journal of Anatomy 208: 753–768. 16761976 10.1111/j.1469-7580.2006.00558.x PMC2100235

[89] Burke D, Hagbarth KE, Skuse NF (1978) Recruitment order of human spindle endings in isometric voluntary contractions. Journal of Physiology-London 285: 101–112. 10.1113/jphysiol.1978.sp012560 PMC1281745 154562

[90] Kearney RE, Hunter IW (1982) Dynamics of human ankle stiffness: variation with displacement amplitude. Journal of Biomechanics 15: 753–756. 7153228 10.1016/0021-9290(82)90090-2

[91] Hunter IW, Kearney RE (1982) Dynamics of human ankle stiffness: variation with mean ankle torque. Journal of Biomechanics 15: 747–752. 7153227 10.1016/0021-9290(82)90089-6

[92] Loeb GE, Brown IE, Cheng EJ (1999) A hierarchical foundation for models of sensorimotor control. Experimental Brain Research 126: 1–18. 10333003 10.1007/s002210050712

