# Supplementary material for: Spinal Mechanisms May Provide a Combination of Intermittent and Continuous Control of Human Posture: Predictions from a Biologically Based Neuromusculoskeletal Model
Source: PLoS Comput Biol. 2014 Nov 13;10(11):e1003944. doi: 10.1371/journal.pcbi.1003944 (PMC4230754; doi:10.1371/journal.pcbi.1003944)
Supplement: Supporting Information S1 — Frequency response of muscle model. This supporting information file provides additional validation data for the muscle model. (PDF) [file pcbi.1003944.s001.pdf]

Supplemental Material

Frequency response of muscle model

The aim of this Supplemental Material is to provide supporting data for the modelling approach used to represent the dynamics of the musculotendon units (MTUs). Experimental data from the Soleus (SO) muscle of healthy humans were reported in [1], and, to our knowledge, no similar data have been obtained for the other leg muscles. Therefore, only the SO muscle model was evaluated and simulation results were compared with human experimental results.

The simulation protocol followed the experimental procedures reported in [1, 2], so that the frequency response of the muscle was estimated by applying intramuscular electrical stimulation. Short-duration electrical pulses (1 ms) were applied at the input of the MTU model. The train of pulses was modelled as a fourth-order homogeneous Gamma point process with a mean intensity equal to 7 Hz. The pulse amplitude was adjusted so as to evoke a force equivalent to 10% of the maximum muscle force. Ankle angle and, consequently, mean MTU length were maintained constant (isometric condition) along the simulation.

Figure 1 shows a 6-s duration sample of input (electrical stimuli) and output (muscle torque) variables. The simulation was sufficiently long (30 s) to allow a reliable estimate of muscle dynamics. Spectral analysis was performed to estimate the frequency response (Equation (1)) and coherence function between input and output (Equation (2)) was calculated to evaluate the linearity of the system [1–3].

$$G(j\omega) = \frac{S_{xy}(j\omega)}{S_{xx}(j\omega)} \tag{1}$$

in which, $S_{xy}(j\omega)$ is the input-output cross-spectrum; and $S_{xx}(j\omega)$ is the input auto-spectrum.

$$\gamma^2(j\omega) = \frac{|S_{xy}(j\omega)|^2}{S_{xx}(j\omega).S_{yy}(j\omega)} \tag{2}$$

in which, $S_{yy}(j\omega)$ is the output auto-spectrum.

The frequency response of SO MTU is shown in Figure 2A. Within a 20-Hz frequency band the muscle frequency response (black line) may be well adjusted by a critically-damped second-order linear system (red line) with a cut-off frequency of ∼2 Hz. Coherence function is approximately 1 within the frequency band evaluated, reinforcing that muscle dynamics is approximately linear for a frequency range of interest. This dynamical behaviour of the MTU model is quite similar to that reported by [1] for the SO muscle.

References

1. Bawa P, Stein RB (1976) Frequency-response of human soleus muscle. Journal of Neurophysiology 39: 788–793.

2. Mannard A, Stein RB (1973) Determination of the frequency response of isometric soleus muscle in the cat using random nerve stimulation. Journal of Physiology 229: 275–296.

3. French AS (1973) Averaging of neurophysiological data prior to frequency domain analysis. Computer Programs in Biomedicine 3: 113–122.

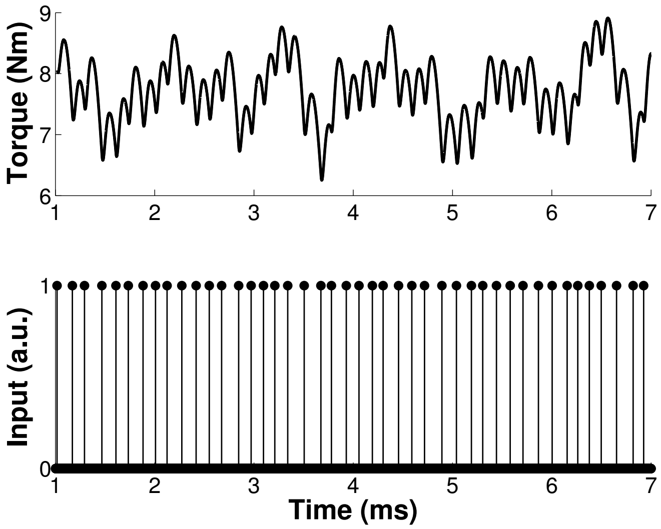

Figure 1. Input and output signals used to estimate the MTU model's frequency response. The upper graph shows the muscle torque produced in response to the random electrical stimulation (lower graph). To improve the visualisation, these graphs show only 6 s of simulated data (whole simulation lasts 30 s).

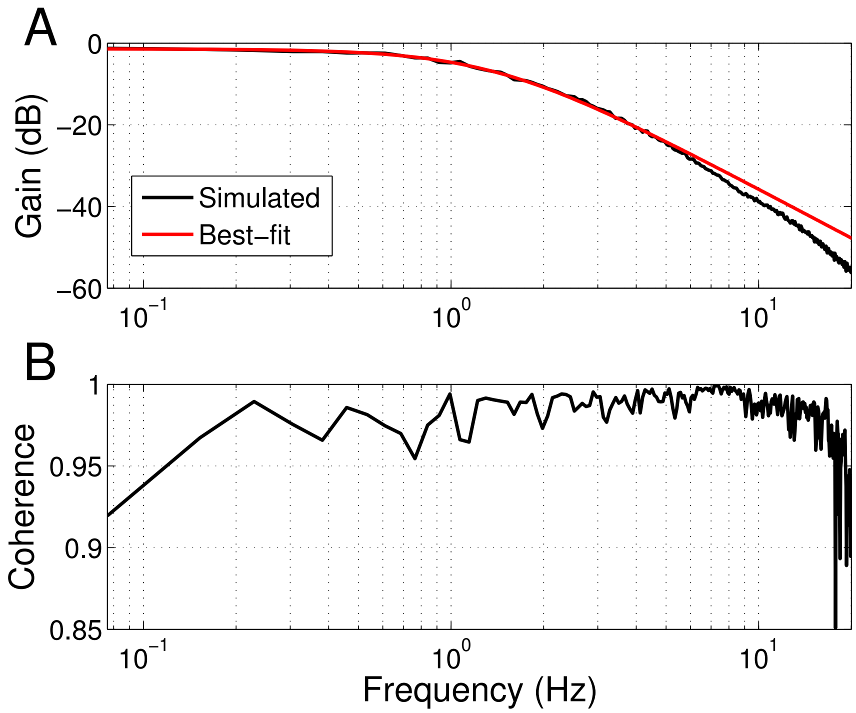

Figure 2. MTU model's frequency response and coherence function. (A) Magnitude of the frequency response estimated by spectral analysis (black curve). The red line represents the least-squares fitting of simulated data by a second-order critically-damped system with a natural frequency of ~ 2. (B) Coherence function shows that the input-output relation is approximately linear ($\gamma^2(j\omega) \approx 1$) for the frequency range evaluated (0-20 Hz).
